# Discovery of functionally distinct anti-C7 monoclonal antibodies and stratification of anti-nicotinic AChR positive Myasthenia Gravis patients

**DOI:** 10.3389/fimmu.2022.968206

**Published:** 2022-09-05

**Authors:** Eleonora Lekova, Wioleta M. Zelek, David Gower, Claus Spitzfaden, Isabelle H. Osuch, Elen John-Morris, Lasse Stach, Darren Gormley, Andrew Sanderson, Angela Bridges, Elizabeth R. Wear, Sebastien Petit-Frere, Michael N. Burden, Richard Priest, Trevor Wattam, Semra J. Kitchen, Maria Feeney, Susannah Davis, B. Paul Morgan, Eva-Maria Nichols

**Affiliations:** ^1^ Immunology Research Unit, GlaxoSmithKline Research & Development (GSK R&D), Stevenage, United Kingdom; ^2^ Division of Infection and Immunity and Dementia Research Institute, Systems Immunity Research Institute, School of Medicine, Cardiff University, Wales, United Kingdom; ^3^ Medicinal Science and Technology, Biopharm Discovery, GlaxoSmithKline Research & Development (GSK R&D), Stevenage, United Kingdom; ^4^ Medicines, Science and Technology, Protein Cellular and Structural Sciences (PCSS) Structural and Biophysical Sciences, GlaxoSmithKline Research & Development (GSK R&D), Stevenage, United Kingdom; ^5^ Medicines, Science and Technology, Protein Cellular and Structural Sciences (PCSS) Protein and Cellular Sciences, GlaxoSmithKline Research & Development (GSK R&D), Stevenage, United Kingdom

**Keywords:** myasthenia gravis, autoantibodies, terminal pathway, MAC assembly, complement C7, myasthenia gravis patient stratification, complement therapy

## Abstract

Myasthenia Gravis (MG) is mediated by autoantibodies against acetylcholine receptors that cause loss of the receptors in the neuromuscular junction. Eculizumab, a C5-inhibitor, is the only approved treatment for MG that mechanistically addresses complement-mediated loss of nicotinic acetylcholine receptors. It is an expensive drug and was approved despite missing the primary efficacy endpoint in the Phase 3 REGAIN study. There are two observations to highlight. Firstly, further C5 inhibitors are in clinical development, but other terminal pathway proteins, such as C7, have been relatively understudied as therapeutic targets, despite the potential for lower and less frequent dosing. Secondly, given the known heterogenous mechanisms of action of autoantibodies in MG, effective patient stratification in the REGAIN trial may have provided more favorable efficacy readouts. We investigated C7 as a target and assessed the *in vitro* function, binding epitopes and mechanism of action of three mAbs against C7. We found the mAbs were human, cynomolgus monkey and/or rat cross-reactive and each had a distinct, novel mechanism of C7 inhibition. TPP1820 was effective in preventing experimental MG in rats in both prophylactic and therapeutic dosing regimens. To enable identification of MG patients that are likely to respond to C7 inhibition, we developed a patient stratification assay and showed in a small cohort of MG patients (n=19) that 63% had significant complement activation and C7-dependent loss of AChRs in this *in vitro* set up. This study provides validation of C7 as a target for treatment of MG and provides a means of identifying patients likely to respond to anti-C7 therapy based on complement-activating properties of patient autoantibodies.

## Introduction

The complement system plays an important role in innate and adaptive immune response and tissue homeostasis and is broadly involved in both common and rare diseases (1). Activation of the complement system through one of three pathways, alternative, lectin and classical, results in formation of a C3 convertase, cleaving C3 into C3a and C3b. The latter facilitates clearance of microorganisms and immune complexes through opsonization as well as initiating formation of the C5 convertase on the cell surface. Cleavage of C5 by C5 convertases initiates the terminal pathway and results in generation of C5a, a potent anaphylatoxin and C5b, which rapidly binds C6, C7, C8 and multiple molecules of C9 to form the membrane attack complex (MAC) (2). The MAC mediates lysis of microorganisms in infection and tissue damage and inflammation in disease (2, 3).

The 2007 FDA approval of Eculizumab (Soliris, Alexion Pharmaceuticals), an anti-C5 monoclonal antibody, for the treatment of Paroxysmal Nocturnal Hemoglobinuria (PNH), presented a significant milestone in the field of complement therapeutics (4, 5). Treatment with Eculizumab requires high, bi-weekly i.v. doses of 1200mg for the treatment of PNH and atypical Hemolytic Uremic Syndrome (aHUS) (4), with an annual per-patient cost of ~$400,000. While many therapeutic concepts and modalities have been explored across the entire complement cascade, relatively few have progressed into clinical development and the terminal pathway remains a focus point (6). Notably, very few approaches have explored other terminal pathway targets, despite the potential for lower, less frequently administered doses. Three C6 antibodies, including Regenemab, CP010 (unpublished pre-clinical candidate, Regenesance), have been described and more recently, a panel of potent C7 and C5b7 inhibitors was described that highlights the potential of lower dosing compared to targeting C5 (7, 8). An important consideration, not specific for anti-complement therapies, but relevant for all high-priced drugs that have potentially serious side-effects that need to be carefully managed, is the need for mechanism-based patient stratification to identify those patients who have a high likelihood of an adequate response.

In 2017, Eculizumab was also approved for the treatment of refractory Myasthenia Gravis (MG), an autoantibody-mediated disease characterized by disrupted cholinergic transmission due to decreased numbers of acetylcholine receptor (AChR) at the neuromuscular junction (NMJ), resulting in weakness and lack of muscular control (9). Before the approval, treatment options for patients were limited to general immune-suppressive drugs and off-label use of Rituximab for B cell depletion (10). Autoantibodies against the muscle nicotinic AChR are detectable by radioimmunoassay in 80 - 90% of MG patients and contribute directly to the MG pathology. In autoantibody-mediated disease, complement activation is driven through Fc effector function of the autoantibodies. The classical pathway can be effectively activated by immune-complex associated IgA, IgM and IgG (2). The importance of complement activation, *via* autoantibodies of the IgG1 and IgG3 subtypes, is well documented in MG (11–13). The serum AChR autoantibody titers in different patients do not correlate with disease severity, which is likely due to the heterogeneity of antibodies and their effector functions, and antibodies have been shown to reduce the number of AChR by at least three mechanisms: endocytosis/degradation mediated *via* antibody receptor crosslinking, functional blockade of acetylcholine binding sites, and complement-mediated AChR loss (14–16). Autoantibody-mediated complement deposition and tissue damage, leading to AChR loss, appears to be one of the main pathological mechanisms as demonstrated by the efficacy of Eculizumab in a phase 2 trial in AChR-antibody positive patients with generalized MG (17). However, the primary efficacy endpoint was missed in the phase 3 REGAIN study (NCT01997229), assessing the safety and efficacy of Eculizumab in anti-AChR antibody-positive refractory generalized myasthenia gravis (13). A possible explanation for this could be that patients were not stratified.

Here, we describe the *in vitro* and *in vivo* characterization of a new anti-C7 monoclonal antibody and provide insights into tractable, functionally relevant epitopes on C7. We show that the antibody ablates disease in rodent MG models and describe a novel method for patient stratification that highlights the heterogeneity of complement-dependence in MG and enables categorization of nicotinic AChR autoantibody positive patients according to complement-dependent loss of AChR.

## Materials and methods

### Anti-C7 antibody generation and scale up and purification of TPP1820

#### Immunizations and antibody discovery

All animal studies were ethically reviewed and carried out in accordance with Animals (Scientific Procedures) Act 1986 and the GSK Policy on the Care, Welfare and Treatment of Animals. Transgenic mice expressing a human V-gene repertoire, were immunized with human C7 protein purified from normal human serum (Complement Technology, Inc). B-cells enriched from the spleen and lymph node tissues were used for hybridoma fusion and direct single B-cell sorting. For the sorting cells were stained with a combination of fluorescently labelled antibodies against B cell markers. Memory and plasma blast B cells were labelled with B220-PECy7, IgM-BV605 and CD43-FITC, plasma cells with B220-PECy7 and CD138-PE. Contaminating cells were excluded by gating out cells positive for CD3, CD93, CD11c, Ter-119 and Gr1, all conjugated to PerCPCy5.5. Antigen specific B-cells and CD138+ plasma cells were single cell sorted using the BD FACS Aria III (Becton Dickinson). To identify C7 binding memory or plasma blast cells the cells were incubated with a biotinylated version of the human protein isolated from human plasma. C7 binding was visualized using streptavidin-PE and streptavidin-APC. cDNA was synthesized from the sorted B-cells and used for V-gene amplification by PCR. Cognate VH and VL chains were then cloned into the Adimab yeast-based platform (Adimab, LLC) and clonal yeast populations with concomitant HC and LC expression and human C7 binding were isolated by FACS, expressed and purified. Antibody clones derived from both the hybridoma and B-cell sorting methods were characterized and selected based on their binding affinity, inhibitory potency in the classical pathway hemolysis assay and epitope diversity.

#### Antibody optimization by generation and selection of random mutagenesis libraries

Affinity maturation libraries were built for the selected, functional anti-C7 antibodies by diversifying each of the complementary determining regions (CDRs) 1, 2, and 3 of the heavy- and light-chain variable region (VH and VL) genes. Random mutations restricted to the CDRs were introduced by splice-overlap-extension (SOE) PCR using degenerate oligonucleotides synthesized with mixtures of nucleotide bases with a bias towards the wild-type nucleotide. Antibodies were selected from the affinity maturation libraries using the Adimab platform according to the protocols developed by Adimab, LLC. Human C7 protein was biotinylated *via* amine coupling, magnetic bead selections were performed using streptavidin beads from Miltenyi (MACS^®^) and FACS selections were performed on the BD FACS Aria III (Becton Dickinson). Cells from the yeast maturation libraries which showed improved antigen binding over the parental antibodies were bulk sorted and plated on agar plates for single colony isolation and sequencing.

#### TTP1820 scale up and purification

The monoclonal antibodies were obtained by transient expression in HEK293-6E cells. Supernatants were collected after 10 days, sterile filtered and the antibodies affinity purified using MabSelect SuRe columns on the Akta Xpress system. Purity and integrity of the purified mAbs was confirmed by analytical size exclusion chromatography and SDS-PAGE. Endotoxin levels were confirmed to be below 0.75 EU/mL using an EndoSafe endotoxin reader (Charles River Laboratories).

### Affinity measurement by surface plasmon resonance

Surface plasmon resonance experiments were performed on a Biacore 8K instrument (Cytiva) using HBS-EP+ (Teknova) at pH 7.4 as a running buffer. For the SPR chip, Protein A (Pierce) was immobilized on a CM5 chip (Cytiva) using a Biacore amine coupling kit (Cytiva) according to the manufacturer’s instructions. Multi-cycle kinetics experiments were run as follows. Antibodies were diluted in running buffer to 0.5μg/ml and captured on the sample flow cells of all channels at a flow rate of 10 μL/min for 60s. Antigen was flowed over both flow cells of all channels at a flow rate of 30μl/min for 180s followed by a 600s dissociation time. The chip surface was regenerated between cycles using 50mM NaOH for 30s at a flow rate of 30μL/min. Antigen concentrations used were 0, 0.39, 1.56, 6.25 and 25nM. The data was analyzed in the Biacore analysis software to a 1:1 binding model using local Rmax and double referencing. Off-rates fitted as slower than 1x10^-5^ 1/s and too slow to be fitted reliably were manually adjusted to 1x10^-5^ 1/s. Antigens used were human complement C7 (Complement Tech) and cyno complement C7 purified from cynomolgus serum (Seralab).

### Hemolysis assays

Sheep erythrocytes (TCS Biosciences) were prepared for assay with gentle washing in CFD (Complement Fixation Diluent, Oxoid Ltd) followed by sensitization with a complement fixation antibody (Amboceptor, Siemens Healthcare) for 30min at 37°C. Control antibodies included were an in-house anti-C5 and disabled anti-C5 antibody (both isotype matched with test mAbs, human Fc-disabled IgG1κ) for human serum and a mouse anti-C7 mAb (Quidel), mouse IgG1κ was used for the rat serum, in addition to the isotype control for the test antibodies. There was no cyno cross-reactive positive control available at the time of the experiments, therefore only the test antibody isotype control was included. Serial dilutions of test antibodies, along with positive and negative control antibodies as above, were prepared in CFD and 50μL added to the wells of a 96-well U-bottomed assay plate (Greiner Bio-One). Pooled normal human serum (or rat serum/cyno serum) was diluted in CFD to a concentration previously determined to elicit 80% lysis of sheep erythrocytes and 50μL were added to the wells of the assay plate containing the serially diluted test molecules. 50μL of diluted serum was also added to wells containing an equal volume of CFD to determine maximum complement induced lysis. The plate was then incubated on ice for 30min. Following incubation, 50μL of sensitized sheep erythrocytes were added to all wells of the assay plate and also to wells containing 100μL of water only (to give 100% lysis control) and wells containing 100μL of CFD only (to give 0% lysis control). The plate was then incubated at 37°C for 30 min to allow complement mediated lysis of the cells to take place. Following lysis, the plate was centrifuged at 500g for 3 min and supernatants transferred to a Maxisorp 96-Well Flat-Bottom Assay Plate (Nunc). Absorbance was measured at 405nm on a Molecular Devices SpectraMax M5 plate reader. The percentage hemolysis was calculated as = 100 x (Test sample at 405nm - A405nm 0% lysis control)/(A405nm 100% lysis control - A405nm 0% lysis control). Dose response curves for the test and control antibodies were derived in GraphPad Prism (v 5.01) using a four-parameter logistic curve fit and EC50 values determined.

### HDX-MS

Hydrogen Deuterium Exchange Mass Spectometry (HDX-MS) was used to determine the C7 binding epitopes of TPP1657, TPP1653 and in the case of TPP1820, the parental molecule TPP1651 was included as TPP1820 was derived later through affinity maturation. Based on our previous experience the epitope in daughter clones does not change as a result of our affinity maturation process, protection patterns tend to be more pronounced. Full experimental details, method and supporting experimental data are located in **Supplementary File 1** which contains **Table S1** (HDX Experimental Data Summary), **Table S2** (Table of peptides with statistically significant protection) and **Figure S1** (Sequence coverage and redundancy), **Figure S2** (Differential fractional uptake and time course data vs. peptide ID), **Figure S3** (Woods plots) and **Figure S4** (Volcano plot analysis).

#### MAC assembly and elucidating anti-C7 antibody mechanism of action using Bio-Layer Interferometry (BLI) technology

BLI experiments were performed on an OctetRed384 instrument (Fortebio) using phosphate buffer saline IgG free (PBSF) buffer. Anti-mouse capture (AMC) biosensors (Fortebio) were equilibrated in PBSF (10 min shaking 200xg) then 133nM anti-C6 monoclonal antibody (Quidel) or 133nM mouse IgG1,κ isotype control (GlaxoSmithKline) were loaded onto the AMC biosensors (600s). Following a PBSF wash step (30 seconds), 100nM of C5b6, C7, C8 and C9 (Complement Technologies) were added to the AMC biosensors sequentially (time varied between 300-800s depending on the protein being assessed). PBSF only controls were included for each protein addition step of forming the complex and represent the negative controls. To determine whether the anti-C7 mAbs prevent C5b6:C7 or C7:C8 interactions binding of C7 in the presence or absence of 2nM or 200nM anti-C7 antibody to previously captured C5b6 biosensors was evaluated (800 seconds). Subsequent steps then sequentially assessed the ability of C7, C8 or C9 complement proteins to bind to the AMC biosensors (800s). In all experiments a PBSF buffer only control, representing a reference biosensor, was included. To subtract background noise, values for the reference biosensor was subtracted from the sample biosensor values. When elucidating anti-C7 antibody method of action, all traces were aligned to the beginning of the addition of C7 step.

### Passive experimental autoimmune Myasthenia Gravis in rats

To measure *in vivo* effects and prophylactic therapeutic efficacy of TPP1820, Lewis rats (150 – 180g) were obtained from Charles River Laboratories (Edinburgh, UK) and allowed to acclimatize for one week. Disease was then induced by subcutaneous administration of anti-Acetylcholine receptor (AChR) mAb35 at 1mg/kg in PBS. MAb35 binds the main immunogenic region of AChR, activating complement and damaging the neuromuscular junction endplates, causing severe muscle weakness. Animals were assessed frequently post-disease initiation for weight loss and clinical symptoms (0, no disease; 1, loss of tail tone and reduced grip strength in back legs; 2, loss of grip in front legs; 3, hind limb weakness and wasting; 4, loss of grip and hind limb paralysis; 5, moribund). The grip strength was assessed by allowing the rat to grip a standard gridded metal cage-top with its rear or forepaws then gently lifting the rat – a healthy rat lifts the lid (0), a weakened rat lifts but loses its grip (1,2), a paralysed rat cannot grip at all (3,4). mAb35-injected rats were split into two groups: Group 1 (n = 8) was treated with mAb TPP1820 at 40 mg/kg dose (standard dose for anti-complement mAb used in animal models, comparable to bolus dose of ~20mg/kg of Eculizumab for initiation of therapy) given by intraperitoneal injection at time zero. Group 2 (n = 8) received an isotype control antibody GRITS 53541 (control EAMG) at the same time, route and dose. All animals were humanely killed by CO_2_ asphyxiation either when weight loss exceeded 20% of starting weight, clinical score exceeded 3 (Home Office license requirements), or at 48 hours (h) post-disease induction. Blood was taken for serum assays at 0, 24 and 48h (or at time of euthanasia if earlier). For the therapeutic efficacy of TPP1820, mAb35-injected rats were split into two groups: Group 1 (n = 8) was treated with test mAb TPP1820 at 40 mg/kg dose (standard dose for anti-complement mAb used in rodent models) given by intraperitoneal injection 16h post-induction of EAMG. Group 2 (n = 8) received an isotype control antibody (GRITS 53541) at the same times, routes and doses. Rats were humanely killed at the end point of the experiment at 48h post-induction, or earlier if clinical score exceeded 3 or weight loss exceeded 20%. Blood was taken for serum assays at 0, 24 and 48h (or at time of kill if earlier).

All animal studies were ethically reviewed and carried out in accordance with Animals (Scientific Procedures) Act 1986 and the GSK Policy on the Care, Welfare and Treatment of Animals.

### Measurement of plasma TCC in rats

Maxisorp plates (Nunc, Loughborough, UK) were coated with rabbit anti-rat C9 IgG (in house; 10µg/ml in bicarbonate buffer, pH 9.6) at 4°C overnight; wells were blocked (1h at 37°C with 3% BSA in PBS), washed once in PBS containing 0.05% Tween 20 (PBS-T). Standard curves of zymosan-activated normal rat serum (serial dilution series staring at 1 in 5) and rat serum samples diluted 1 in 50 in 1% BSA-PBS, 20mM EDTA were added in duplicate and incubated overnight at 4°C. Wells were washed with PBS-T 3X, then incubated (2h, room temperature (RT)) with monoclonal 12C3 anti- rat/mouse C5b-9 (in house; 5µg/ml in PBS-T). After washing, wells were incubated (1h, RT) with biotinylated donkey anti-Mouse IgG (1:2000 in PBS-T; Jackson ImmunoResearch #715-005-150). After washing, Streptavidin-HRP (1:2000 in PBS-T; R&D Systems, # 890803) was added and incubated 30 minutes at RT. After washing, plates were developed using o-phenylenediamine dihydrochloride (SIGMAFAST; Sigma-Aldrich, St Louis, MO) and absorbance (492 nm) was measured. Protein concentrations (Units/ml) of serum samples were automatically calculated by reference to the standard curve using GraphPad Prism (GraphPad, La Jolla, CA, USA).

### *Ex vivo* hemolysis assay

The assay system comprised optimally antibody-sensitized sheep erythrocytes (ShEA) incubated with rat serum (NRS) dilutions in HEPES-based buffer supplemented with Ca^2+^ and Mg^2+^ cations (assay buffer) (SOP in (18)). Sheep blood was from TCS Bioscience and anti-ShE antiserum (#ORLC25, Siemens Amboceptor) was from Cruinn Diagnostics (Dublin, UK). In an initial *in vitro* assay, a serum dilution was selected to give ~75-90% hemolysis of ShEA at end point (30min at 37°C; typically, 2-5% NRS) in the absence of inhibitor. Test and positive control mAb were then titrated in a broad range of concentrations (in triplicate) into the NRS dilutions (100µL) in the wells of a U-welled microtiter plate and ShEA (100µL) at the appropriate dilution in assay buffer added and incubated for 30min at 37°C. Plates were centrifuged, supernatants removed to a flat-well microtiter plate and absorbances measured spectrophometrically. Percent hemolysis was calculated for each well by comparison with 0 and 100% lysis controls. Hemolytic activity in sera from the experimental animals were tested in essentially the same assay; rat serum dilutions (10-0%, in triplicate, 100µL per well in assay buffer) were made in the wells of a U-welled microtiter plate and ShEA (100µL) in assay buffer were added and incubated for 30min at 37°C. Plates were centrifuged, supernatants removed to a flat-welled microtiter plate and absorbances measured spectrophometrically (A405) and % lysis calculated for each sample.

Hemolytic activity in IgG-depleted NHS was tested as described above but using complement fixation diluent (CFD) (Oxoid Ltd.) rather than HBS buffer. The percentage hemolysis was calculated as = 100 x (A405 nm test sample - A405 nm 0% lysis control)/(A405 nm 100% lysis control - A405 nm 0% lysis control). The NHS hemolysis dose-response curves were plotted in GraphPad Prism v7 and analyzed using a four-parameter logistic curve fit to calculate the EC50 concentrations.

### Plasmid generation

AChRαβ & AChRδϵ plasmids: cDNAs for the α1/β1 and δ/ε subunits of human nAChR were cloned into the dual expression vectors pBiCIH and pBiCIN respectively. pBiCIH and pBiCIN are hygromycin and neomycin resistant derivatives of the bi-cistronic vector pCIN5 (19), modified by insertion of a SV40-promoter-polyadenylation signal cassette to allow expression of a second gene insert. In pBiCIH and pBiCIN, the α1 and δ subunits are expressed from the CMV promoter as bi-cistronic inserts linked to the selectable marker by an IRES element; the β1 and ε subunits are expressed from the SV40 promoter.

Rapsyn plasmid: The pCIP4 plasmid backbone (made internally) contains an IRES element upstream of the puromycin resistance cassette, allowing stable selection of cell lines. The Rapsyn gene (NM_005055) was synthesized at GenScript, then subcloned into pCIP4 using NotI and BamHI restriction sites.

### Transient cell transfection

ARPE-19 CD46, CD55, CD59 KO cells were available as a suitable tool cell line as they are susceptible to complement attack, without the need of blocking antibodies to the above complement regulatory proteins. The cells were cultured in DMEM/F12 + GlutaMAX medium (Invitrogen), supplemented with 10% HI-FBS (Invitrogen), 15mM HEPES (Invitrogen), and 1X MEM-NEAA (Invitrogen) (complete culture medium). Generation of these cells is described in **Supplementary File 2**. Due to the biolicensing terms of the CRISPR reagents, this cell line cannot be made available and a protocol to replicate the effect of complement regulator deficiency is included in **Supplementary File 2**. One day before transfection, the cells were seeded in 96-well black glass-bottom plates (Greiner) at a cell density of 20,000 cells/well in complete culture medium and incubated overnight at 37°C, 5% CO_2_, high humidity. Lipofectamine/DNA lipid complexes were prepared in Opti-MEM™ Medium (Invitrogen) according to the manufacturer’s instructions: 0.2μg total DNA and 0.15μL lipofectamine was used per well. The following plasmids were added at a molar ratio of 1:1:1 (0.055μg pCIP4-huRapsyn, 0.074μg pBiCIH-hnAChRα1β1, and 0.071μg pBiCIN-hnAChRδϵ per well). The culture medium from the wells was aspirated and replaced with lipofectamine/DNA lipid complex, pre-diluted 1 in 10 in complete culture medium. The cells were incubated for 24h at 37°C, 5% CO_2_, high humidity.

### Quantification of AChR expression on transfected cells

The cells were blocked following transfection with 200μL/well of complete culture medium + 1% BSA (staining buffer) for 15 – 30 min at 37°C, 5% CO_2_, high humidity, then stained with 50μL/well Alexa Fluor™ 647 conjugated α-Bungarotoxin (AF647 α-Bungarotoxin; Invitrogen) at 2.5μg/ml in staining buffer and incubated for 30 min at 37°C, 5% CO_2_, high humidity. The cells were washed twice with 200μL/well of PBS and fixed for 10 min at RT with 50μL/well of 10% Formalin (Sigma), washed twice with 200μL/well of PBS and stained with a solution, containing 10 Units/ml Alexa Fluor™ 488 Phalloidin (actin stain, Invitrogen) and 1μg/ml Hoechst 33342 (nuclear stain, Invitrogen). The cells were incubated for 30min at 37°C, 5% CO_2_, high humidity, and washed 2x with 200μL/well PBS. PBS was added to the wells at 100μL/well and images were captured on an InCell 2200 or InCell 6000 imager (GE Healthcare), using a 10X objective. Images were analyzed using Columbus v2.8 (Perkin Elmer). The following image analysis algorithm was used. The fluorescence from the DAPI channel (Hoechst 33342 stain) was used to find the nuclei. The fluorescence from the FITC channel (Alexa Fluor™ 488 Phalloidin stain) was then used to identify the cytoplasm and draw the cell perimeter. Spots of clustered surface AChR were identified within each cell, based on the AF647 α-Bungarotoxin (Cy5) fluorescence, and various spot parameters were calculated. The sum of the spot fluorescence per cell was used to set the threshold for identifying “AChR Pos Cells”. The % positive cells was calculated by dividing the number of positive cells by the total number of cells, multiplied by 100. The data was plotted in GraphPad Prism v7.05.

### Human sample collection

Myasthenia Gravis patient plasma samples were purchased from Tissue Solutions (Glasgow, UK). These patients were confirmed as anti-nicotonic AChR positive based on a autoanitobdy titre determined by immunoassay (see **Table S3** for patient details).

Pooled control plasma was prepared from blood obtained from healthy volunteers at GSKs internal blood donation unit. 100mL of EDTA anti-coagulated blood was obtained per donor from six donors in total. The blood was centrifuged for 10 min at 2000xg, 4°C and the plasma supernatants were transferred to clean 50mL falcon tubes which were centrifuged again for 5 min at 2000 x g, 4°C. All plasma supernatants from all donors were pooled into a sterile reservoir, aliquoted into sterile Eppendorf tubes, and stored at -80°C.

Pooled normal human serum (NHS) was prepared from blood obtained from healthy volunteers at GSKs internal blood donation unit. Blood was collected from 10 healthy volunteers in serum separator tubes (S-Monovette^®^ 7.5ml Z-Gel, Sarstedt) and processed to preserve complement activity. The blood was left to clot at RT for 20 – 30min, then the tubes were immersed in ice water to contract the clots and centrifuged for 10min at 2000xg at 4°C. The serum supernatants from all tubes were pooled into a 50 ml Falcon tube. The tube was centrifuged for 5min at 2000xg, 4°C. The supernatant was transferred into a fresh reservoir, aliquoted and frozen immediately at -80°C.

The human biological samples described were sourced ethically and their research use was in accord with the terms of the informed consents under an IRB/EC approved protocol.

### ELISA for quantification of patient autoantibody titers in-house

The Anti-Acetylcholine Receptor ELISA (IgG) kit from Euroimmun (catalogue number EA 1435-9601 G) was used for quantification of patient autoantibody titers in-house. The plasma test samples were thawed at RT and diluted 1:26 in sample buffer. The 1:26 sample dilutions were considered as neat samples on the standard curve, and controls were also diluted 1:26. Some of the samples which had higher titers were further diluted and dilution factor from this step was used when calculating the sample concentrations from the standard curve. The absorbance was read at 450 nm and 650 nm on a PHERAstar FSX. The data analysis was carried in the PHERAstar FSX MARS Software v3.20 R2: the blank and 650 nm values were subtracted from all wells and a standard curve was plotted and analyzed using a four-parameter logistic curve-fit. The unknown sample concentrations were calculated using the four-parameter logistic equation and multiplied by the dilution factor (if applicable). Each sample was tested in three independent experiments and the mean of the three experiments was calculated.

### Immunoglobulin (Ig)-depletion of normal human serum

The pooled NHS was depleted of immunoglobulins using Protein A/G agarose (Thermo Scientific) in order to remove natural antibodies that may bind to the cells and cause non-specific MAC deposition. 15mL Protein A/G agarose resin was used per 3.5mL NHS. The resin was washed three times with 35mL PBS and placed on ice. The NHS was thawed in a water bath at 37°C, mixed with the resin and incubated for 30min in a 4°C cold cabinet on a roller mixer. The NHS/resin suspension was centrifuged for 2min at 1000xg, 0°C. The supernatant was aliquoted in Eppendorf tubes and immediately frozen at -80°C. Except for the assay validation data, all MG experiments reported used Ig-depleted NHS as source of complement.

### MSD assay for human immunoglobulin

To confirm whether immunoglobulin depletion was successful, NHS and Ig-depleted NHS were tested for IgG, IgA, and IgM levels using a commercial immunoassay kit (Human/NHP Isotyping Panel 1 kit, Meso Scale Discovery). NHS samples were assayed at dilutions ranging from 1/20 – 1/1,562,500 (1/5 serial dilution intervals) according to the manufacturer’s instructions. The data was analyzed using the MSD Discovery Workbench Software v4.0.12. The background (Blank values) was subtracted from all samples/standards (ECL Signal - Blank). A standard curve was generated by plotting the “ECL Signal – Blank” values against the concentration and analyzed using a four-parameter logistic curve-fit, including a 1/Y2 weighting function. The unknown sample concentrations were calculated using the four-parameter logistic equation and multiplied by the dilution factor where applicable.

### Patient autoantibody binding assay

The transfected cells were blocked with 200μL/well of complete culture medium + 1% BSA and a 1/40 dilution of TruStain FcR block (Biolegend) for 30-60min at 37°C. MG and control plasma samples were thawed and incubated in a heat-block for 30-45min at 56°C to heat-inactivate complement. The heat-inactivated samples were diluted 1/10 in complete culture medium and added to the blocked wells at 50μL/well. The plates were incubated for 30min at 37°C, 5% CO_2_, 95% humidity. The wells were washed twice with 200μL/well of PBS and stained with 2.5μg/ml AF647 α- Bungarotoxin, combined with 20μg/ml of either Cy3 Donkey Anti-human IgG, Fcγ specific, or Cy3 Donkey Anti-human IgM, Fc5μ specific, or Cy3 Donkey Anti-human IgA, α chain specific (all Jackson ImmunoResearch). The plates were incubated for 30min at 37°C, 5% CO_2_, high humidity and washed twice with 200μL/well of PBS. The cells were fixed for 10min at RT with 50μL/well of 10% Formalin (Sigma), then washed and stained with 1μg/ml Hoechst 33342 (nuclear stain) + 10 Units/ml AF488 Phalloidin (actin stain) for 30min at 37°C, 5% CO_2_, high humidity. The wells were then washed, filled with 100μL/well PBS and imaged on an InCell 6000 imager (GE Healthcare), using a 10X objective. Images were analyzed using Columbus v2.8 (Perkin Elmer). The following image analysis algorithm was used. The fluorescence from the DAPI channel (Hoechst 33342 stain) was used to find the nuclei. The fluorescence from the FITC channel (Alexa Fluor™ 488 Phalloidin stain) was then used to identify the cytoplasm and draw the cell perimeter. Spots of clustered surface AChR were identified within each cell, based on the AF647 α- Bungarotoxin (Cy5) fluorescence, and various spot parameters were calculated. The sum of the spot fluorescence per cell was used to set the threshold for identifying “AChR Pos Cells” (Cy5) and “Anti-human Immunoglobulin Pos Cells” (dsRed). The % positive cells was calculated by dividing the number of positive cells by the total number of cells, multiplied by 100. The same calculation was used for the % negative cells but using the number of negative cells. To correct for non-AChR specific immunoglobulin binding, the % Immunoglobulin positive cells within the AChR negative cell population were subtracted (background corrected). The average background-corrected values from three independent experiments (except for MG patient 1 where there was only sufficient sample for one experiment) were plotted in GraphPad Prism v7.03, where samples were classed into the following four categories, based on the average percentage of positive cells: between 0% - 15% (-), between 15% - 40% (+), between 40% - 65% (++), between 65% - 100% (+++). Statistical analysis was carried out in GraphPad Prism v7.05, using a repeated measures 1way ANOVA without correction, with Dunnet’s multiple comparison test, comparing the means (n=3 experiments) of each plasma sample to the mean of the pooled control plasma sample. For MG Patient 1 (n=1 experiment, 2 replicates) the mean of the two replicates was compared to the mean of the two pooled control plasma replicates from the same experiment using an unpaired t-test.

### Complement-mediated AChR loss and MAC deposition assays

The transfected cells were blocked with 200μL/well of complete culture medium + 1% BSA and a 1/40 dilution of TruStain FcR block for 30-60 min at 37°C. MG and control plasma samples were thawed and incubated in a heat-block for 30-45 min at 56°C to inactivate complement. The heat-inactivated samples were diluted 1/10 in complete culture medium. A 10μg/ml mAb35 (Rat anti-AChR tool Ab, Genetex) solution was also prepared in culture medium. The blocking solution from the plates was aspirated and the diluted MG plasma samples or controls were added to the cells and incubated for 30min at 37°C, 5% CO_2_, high humidity. The wells were washed twice with 180μL/well of PBS and the anti-C7 blocking antibody or the corresponding isotype control antibody (both at 20μg/ml in culture medium) added to the appropriate wells at 50μL/well. Culture medium alone was added to control wells. Ig-depleted NHS aliquots were quickly thawed in a water bath at 37°C and placed on ice prior to dilution to 30% in culture medium immediately before addition to the plates (50μL/well). Culture medium alone was added to control wells. The plates were incubated for 2.5-3h at 37°C, 5% CO_2_, high humidity, washed twice with 180μL/well of PBS and stained with 50μL/well of 2.5μg/ml AF647 α-Bungarotoxin + 10μg/ml polyclonal Rabbit anti-C5b9 antibody (Abcam) in staining buffer (culture medium + 1% BSA) for 30 min at 37°C, 5% CO_2_, high humidity. The wells were washed twice with 180μL/well of PBS and fixed with 50μL/well of 10% Formalin for 10min at RT, the washed again as above. A staining cocktail was prepared, containing 1μg/ml Hoechst 33342 (nuclear stain) + 10 Units/ml AF488 Phalloidin (actin stain) + 1/200 dilution of PE Donkey Anti-Rabbit secondary antibody (Jackson ImmunoResearch) in staining buffer and added to the wells at 50μL/well. The plates were incubated for 30min at 37°C, 5% CO_2_, high humidity (95%), washed twice with 180μL/well of PBS, 100μL/well PBS added, and the plates were imaged on an InCell 6000 imager using a 10X magnification. Images were analyzed using Columbus v2.8 (Perkin Elmer). The following image analysis algorithm was used. The fluorescence from the DAPI channel (Hoechst 33342 stain) was used to find the nuclei. The fluorescence from the FITC channel (Alexa Fluor™ 488 Phalloidin stain) was then used to identify the cytoplasm and draw the cell perimeter. Fluorescent spots of clustered surface AChR (Cy5 fluorescence) and deposited MAC (dsRed fluorescence) were identified within each cell, and various spot parameters were calculated. The sum of the spot fluorescence per cell was used to set the threshold for identifying “AChR Pos Cells” and “MAC Pos Cells”. The % positive cells was calculated by dividing the number of positive cells by the total number of cells, multiplied by 100. The “AChR Pos Cell Population: % MAC Pos Cells” was calculated by dividing the AChR/MAC double positive cells by the total number of AChR positive cells, multiplied by 100. To be able to class patients into different categories based on levels of AChR loss and MAC deposition, a “fold change relative to NHS treatment” ratio was calculated for all treatment conditions to normalize the data from different experiments, by dividing each treatment value by the NHS-treatment value for that plate. For the AChR readout the classification was as follows: 1 – 0.8 (-), 0.8 – 0.6 (+), 0.6 – 0.3 (++). For the MAC readout the classification was as follows: 1 – 1.3 (-), 1.3 – 1.8 (+), 1.8 – 2.3 (++), 2.3 – 3.5 (+++). The % positive cells values were plotted in GraphPad Prism v7.05 and statistical analysis was performed using a repeated measures 1way ANOVA without correction, using Tukey’s multiple comparisons test. For patient categories 1, 2, and 4, the average of all donors within the category was analyzed. For individual donors (except Patient 1), statistical analysis was performed on the average of n=3 experiments, each carried out in duplicate. For MG Patient 1 (category 3) (n=1 experiment, 2 replicates) an unpaired t-test was used to compare the mean of duplicates between conditions.

### Correlation between ELISA anti-AChR titers, IgG cell binding to AChR and MAC deposition

A two-tailed, non-parametric Spearman correlation with 95% confidence interval was calculated in GraphPad Prism v7.05 between the following pairs of sample sets: IgG cell binding to AChR vs. MAC deposition on the AChR positive cells, IgG cell binding to AChR vs. ELISA anti-AChR titres, and ELISA anti-AChR titres vs. MAC deposition on the AChR positive cells. The values used for IgG cell binding were the background corrected fluorescent intensity averages of 3 replicates for all samples, except for MG Patient 1, where the average of two replicates from one experiment was used as there was not enough sample for further repeats. The values used for MAC deposition on the AChR positive cells were the “MG Plasma + NHS” fluorescent intensity averages of 3 replicates for all samples, except for MG Patient 1, where the average of two replicates from one experiment was used as there was not enough sample for further repeats. The values used to calculate the correlations for the ELISA anti-AChR titres were the average of 3 replicates for all samples, except for MG Patient 1, where the titer provided in the supplier datasheet was used as there was not enough sample to re-test in-house.

## Results

### Novel function-blocking anti-C7 mAbs show distinct C7 binding epitopes and mechanism of action

Functional anti-C7 antibodies were discovered by immunization of transgenic mice, that have a diverse human V-gene repertoire, combined with standard hybridoma techniques and B-cell sorting methods. 397 antibodies were screened by Surface Plasmon Resonance (SPR) to measure human C7 binding affinity (Kd), functional blockers were subsequently identified in a single dose classical pathway hemolysis assay (>30). Selected antibodies were grouped according to epitope bins using a sandwich Surface Plasmon Resonance (SPR) based method. The top seven functional, epitope diverse antibodies, were optimized for affinity and potency. Affinity optimization was achieved using the Adimab yeast-based platform, improved binders were selected from mutagenesis libraries generated by introducing random diversity into the CDRs of the heavy- and light-chain-variable genes. A total of 649 sequence unique antibodies were screened by Surface Plasmon Resonance for C7 binding. The optimization campaign delivered a hit panel of high-affinity and high-potency antibodies (with single-digit nanomolar IC50 potencies in the hemolysis assay). The antibodies were purified, stability and biophysical properties evaluated (data not shown) and further characterized functionally as described below.

**Figure 1:**
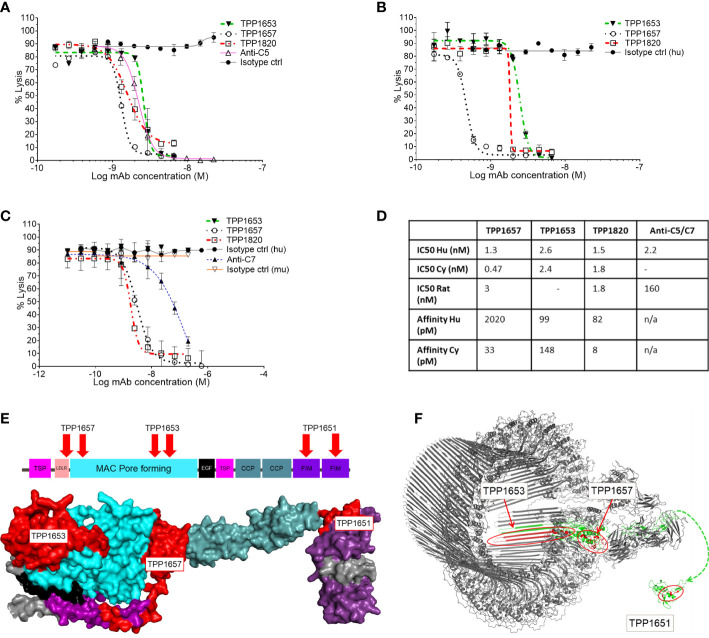
Functional characterization of an anti-C7 monoclonal antibody and HDX-MS epitope mapping. Classical pathway sheep haemolysis assays were used to test the function of TPP1653 (closed triangle), TPP1657 (open circle) and TPP1820 (open square) in normal human serum, comparing to isotype matched active (open triangle) and disabled anti-C5 (closed circle) control antibodies **(A)**. Activity in normal cyno serum was relative to an isotype control for test antibodies only **(B)** and activity in rat was relative to an anti-C7 control and relative human and mouse isotype controls **(C)**. The haemolysis data are representative of several independent experiments. The affinity of the mAbs for human and cyno C7 was measured by SPR **(D)**. **(E)** Diagram of the domain structure of C7 and space filling model of the homologous part of the C6 crystal structure (res. 50-913) representative of the open conformation of C7 (Aleshin et al., 2012; PDB code 3T5O). The model is colour coded by domain. HDX epitopes of TPP1653, TPP1657 and the parent molecule TPP1651 of TPP1820 are coloured red. **(F)** Cryo-EM structure of the membrane attack complex (Menny et al., 2018; PDB code 6H04) and NMR structure of the FIM domain of C7 (Phelan et al., 2009; PDB code 2WCY). The C7 moiety of the complex is shown in green. Significant HDX Protection patterns of TPP1651, 1653 and 1657 were mapped in red. A dashed line linking N693 of the MAC structure to N693 of the first FIM domain indicates the context of the two structures.

A diverse panel of antibodies, that represented four distinct epitope bins determined by SPR, was selected from the hits using binding and affinity data to human and cyno C7. Three representative clones (TPP1657, TPP1653 and TPP1820 or its parental clone TPP1651) were included in the present work. Species cross-reactivity of the three mAbs was assessed using classical pathway hemolysis assays (CP CH50) using normal human, rat and cyno sera. In hemolysis assays using normal human and cyno serum, TPP1657 and TPP1820 showed comparable function with IC50s below 2nM for both species, while TPP1653 had IC50s of 2.6nM and 2.4nM for human and cyno, respectively (**Figures 1A, B, D** **)**. TPP1657 was particularly potent in cyno serum with an IC50 of 0.47nM. TPP1657 and TPP1820 also inhibited rat C7, displaying IC50s of 3nM and 1.8nM, respectively; TPP1653 did not cross-react with rat (**Figures 1C, D** **)**. SPR showed binding of all three mAbs to human and cyno C7 with high affinities in the pM range, with exception of TPP1657 which had an affinity for human C7 off 2nM (**Figure 1D**).

The three mAbs were determined to be in separate epitope bins by competitive ELISA (data not shown). HDX-MS was applied to determine the C7 binding epitopes of TPP1657, TPP1653 and in the case of TPP1820, the parental molecule TTP1651 was included. For this, mAb/C7 complexes or free C7 were deuterium labelled, the reactions quenched, and samples subjected to mass spectrometry. Protected peptides, indicative of binding epitopes on C7, were determined by comparing peptide uptake plots for complexed and free C7 (**Supplementary File 1**). Epitopes for three different domains of human C7 were identified for the mAbs (**Figure 1E**). The protection patterns returned imply that the epitope for TPP1820 is in the C-terminal FIM domains (residues 717-746) (**Figure 1F**). TPP1653 binds to residues 335-362 and 393-408 of the MAC pore forming (MACPF) region (**Figure 1F**). TPP1657 epitope includes the LDLRA and possibly the bottom of the MACPF (residues 90-110 and 175-185) (**Figure 1F**).

**Figure 2:**
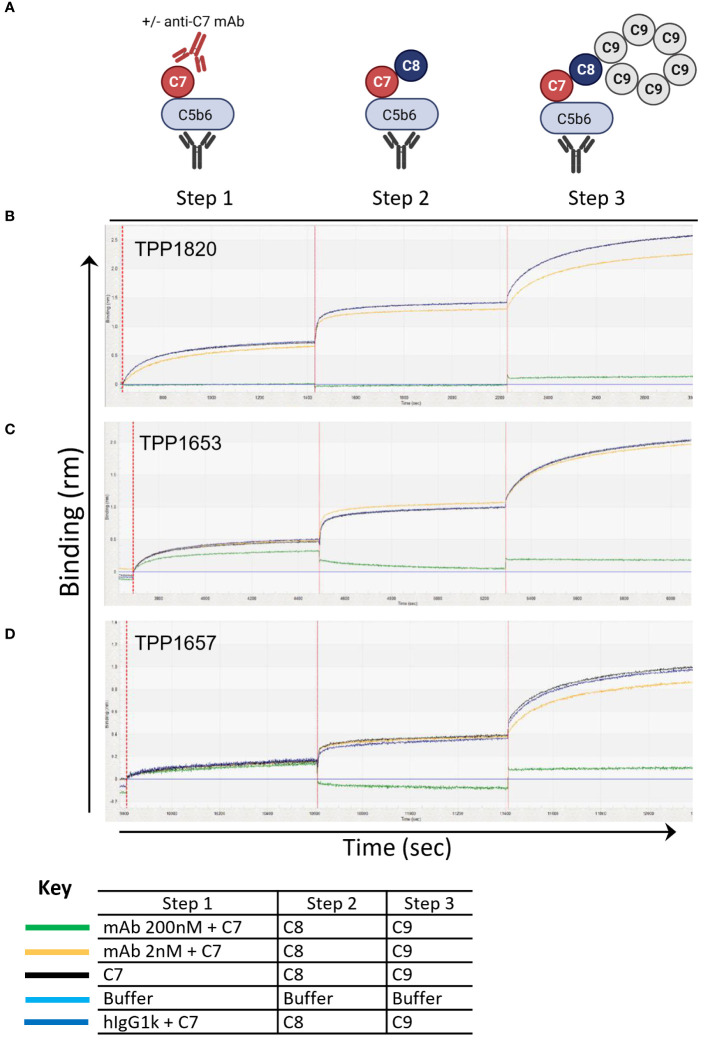
Assembly of the MAC complex using BLI technology – determine anti-C7 antibody mechanism of action. The sensograms demonstrate the assembly of MAC in the presence of absence of anti-C7 antibodies TPP1653, TPP1657 and TPP1820 following C5b6 capture onto AMC sensor (previous capture of anti-C6 mAb and C5b6 binding are not shown). Sensograms for each anti-C7 antibody evaluated are aligned to the C7/anti-C7 antibody addition step and the data has been reference subtracted (reference = αC6 mAb and C5b6 only, lighter blue trace). **(A)** Schematic representation of assembly MAC and evaluating anti-C7 mechanism of action. **(B)** 200nM and 2 nM of antibody TPP1820, **(C)** TPP1653, **(D)** and TPP1657 were pre-incubated with 100nM C7 then the C5b6 captured AMC biosensors were dipped into C7/anti-C7 wells (step 1) before sequential addition into the C8 (step 2) and C9 wells (step 3). Traces were compared to the positive controls with anti-C7 antibody absent or to the hIgG1k isotype control (black and darker blue traces respectively). This figure represents 1 of the 3 experiments conducted.

To further assess the mechanism of action of the mAbs, a bio-layer-interferometry assay (Octet system) was developed to model stepwise assembly of the MAC on AMC biosensors. For this C5b6 was captured on the sensors and dipped sequentially into C7 with an excess of anti-C7 mAb, isotype control or without antibody, followed by C8 and C9 (**Figure 2A**). In this assay, no binding response was measured upon dipping the C5b6 coated sensors in TPP1820/C7 mix demonstrating that the antibody blocked C7 binding into the complex (**Figure 2B**). In contrast, a partial binding response to C7 (~60% of max response in controls) was recorded in presence of 200nM TPP1653, but no further response upon addition of C8 (**Figure 2C**). The TPP1657 binding response was comparable to the control conditions, demonstrating no impact of antibody on complex binding; however, no further response was measured upon addition of C8, showing that the antibody prevented C8 binding the C5b-7 complex (**Figure 2D**).

### TPP1820 is an effective therapeutic for experimental Myasthenia Gravis in rats

**Figure 3:**
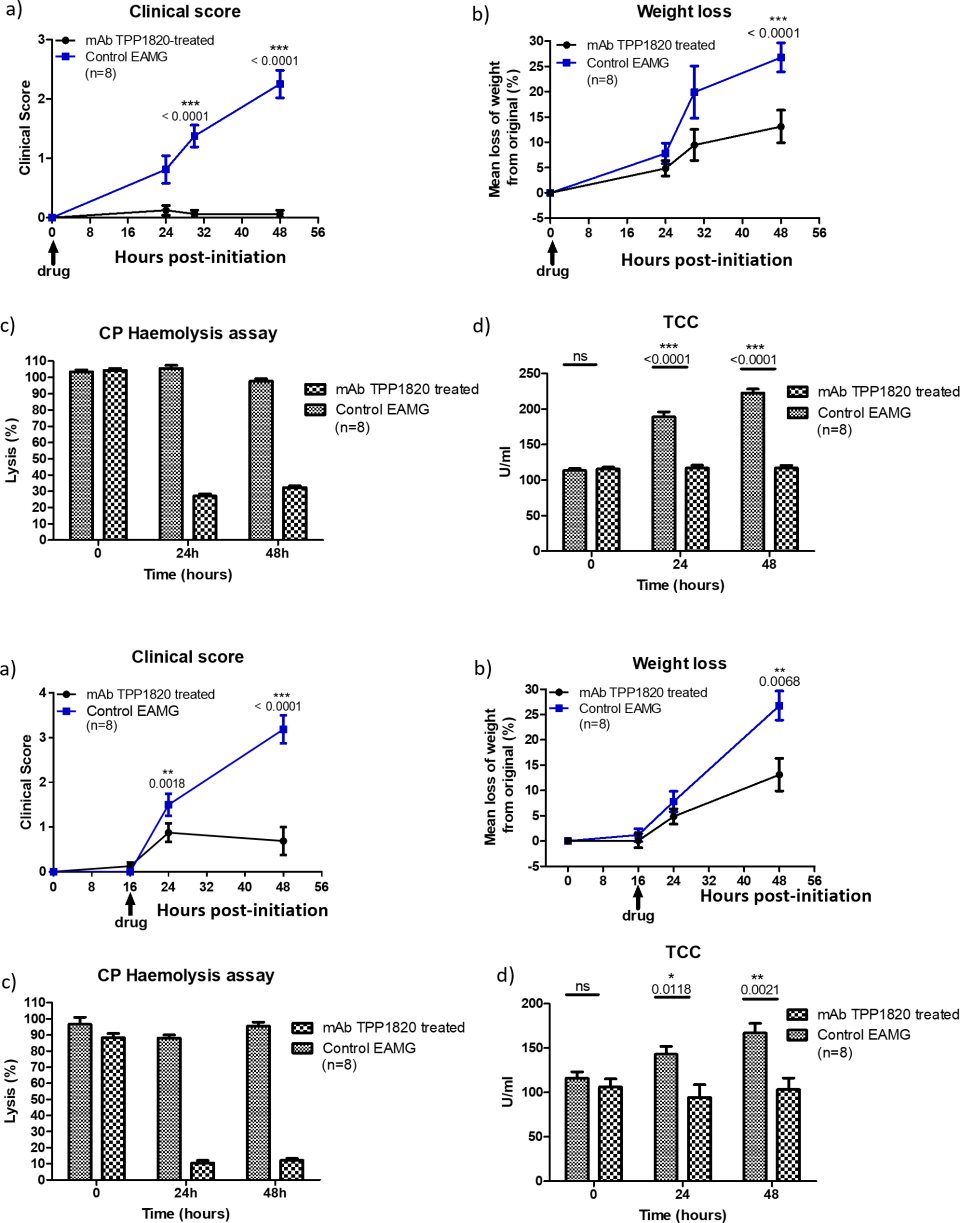
Therapeutic effect of monoclonal antibody (mAb) TPP1820 in experimental autoimmune myasthenia gravis (EAMG). TPP1820 or an isotype control IgG was administered either at the time of EAMG induction (time 0; 3A) or 8 hours after disease induction (3B) with 8 rats in each group in each experiment. Clinical score, assessed as described below, and weight change were monitored (**A**, a) - b) and **B**, a) - b)). In both experiments, control EAMG animals rapidly developed muscle weakness reaching clinical scores of three or four; in contrast, TPP1820-treated rats showed minimal clinical disease across the time course. In both experiments, control animals showed reduced weight gain or even weight loss, while TPP1820-treated animals continued to gain weight over the experiment. All animals were bled pre-induction and at 24 and 48 hours for measurement of serum hemolytic activity and terminal complement complex levels (**A**, c) - d) and **B**, c) - d)). Hemolytic activity remained high in control EAMG animals in both experiments, while TPP1820-treated rats showed markedly reduced hemolytic activity at 24 and 4h hours in both experiments. In control rats, there was an increase in TCC levels at 24 and 48hr; in contrast, TP1820-treated rats showed no increase in TCC levels in either experiment. Differences in TCC levels between the groups were significant at 24 and 48 hours in each experiment. Results are means of determinations from eight TPP1820-treated and eight isotype control-treated EAMG animals in each experiment. Error bars represent SD. Clinical disease was scored as follows: 1. Reduced grip strength in front legs and floppy tail; 2. loss of grip in back legs; 3. loss of grip and hind limb weakness and wasting; 4. loss of grip and hind limb paralysis; 5. moribund. *p <0.05, **p <0.01, ***p <0.001, ns = not significant.

Rats treated with the isotype control mAb, either at the time of disease initiation or 16h post-initiation, developed progressive disease and lost weight as expected in the model. In contrast, rats treated with TPP1820 at the time of induction showed no clinical disease and steady weight gain over the course of the experiments (**Figures 3A**, a) and b)). When treatment with mAb TPP1820 was delayed to 16h post-initiation, early disease was evident at 24h but this was significantly lower than in controls and stabilized with even lower disease scores and significantly greater weight gain compared to controls at 48h (**Figures 3B**, a) and b)). TPP1820 given at 40mg/kg completely inhibited serum lytic activity at 24 and 48h; treatment with the control mAb did not inhibit lytic activity (**Figures 3A**, c), **3B**, c)). Serum levels of the complement activation product TCC were significantly elevated at 24 and 48h in controls compared to TPP1820-treated rats (P<0.0001); TCC levels in TPP1820-treated rats did not change over the time course (**Figures 3A**, d), **3B**, d)).

#### Robust Myasthenia Gravis patient stratification assay reproduces MG disease biology *in vitro* using tool anti-AChR antibody mAb35

**Figure 4:**
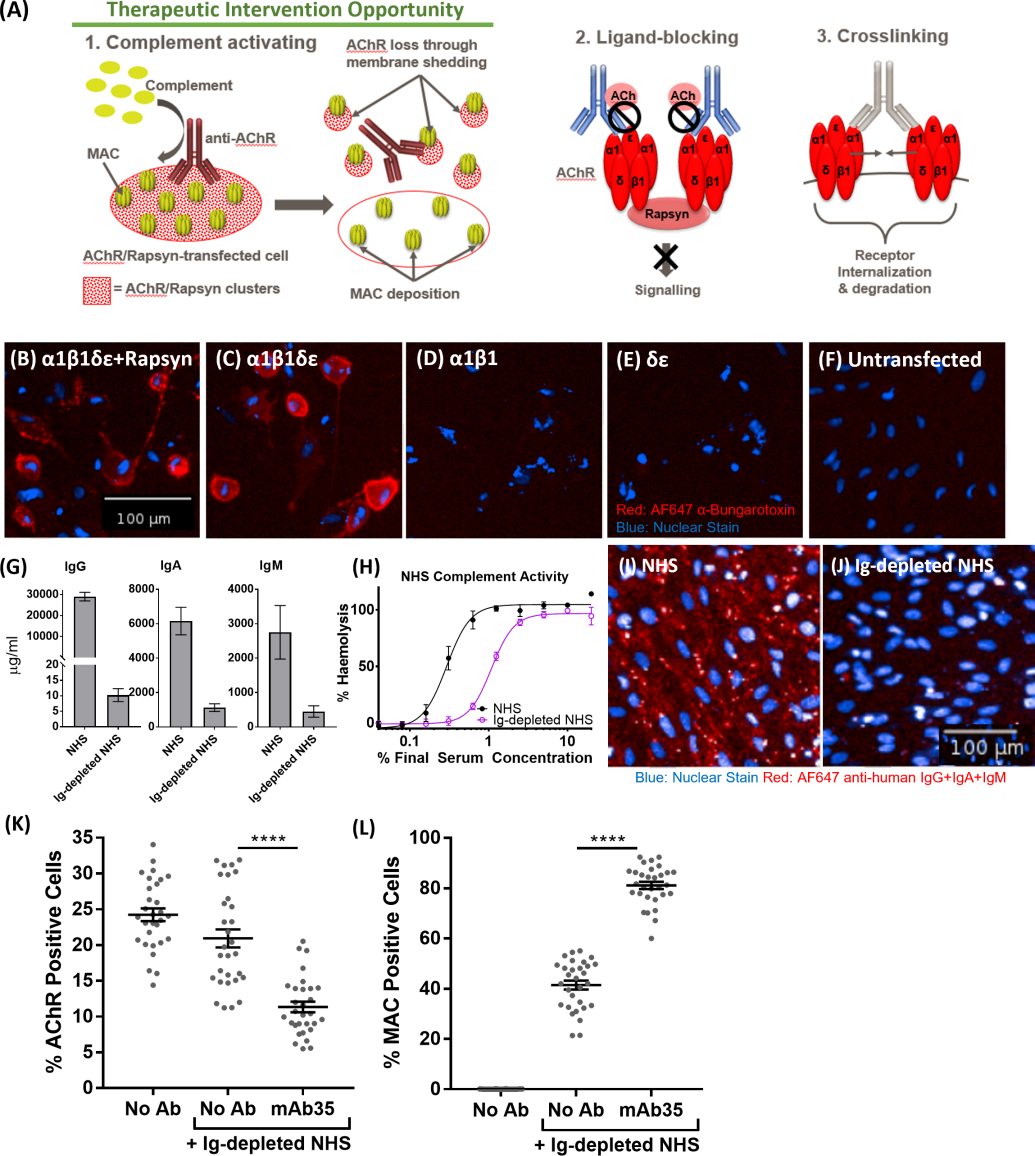
**(A)** Schematic representation of Myasthenia Gravis disease biology driven by anti-AChR autoantibodies. **(B–F)** AF647 α-Bungarotoxin (in red) and nuclear stain (in blue) binding to cells transfected with AChRα1β1δϵ+Rapsyn **(B)**, AChRα1β1δϵ **(C)**, AChRα1β1 **(D)**, AChRδϵ **(E)**, Untransfected control cells **(F, G)** IgG, IgA, and IgM quantification of immunoglobulin-depleted vs. non-depleted NHS. **(H)** Haemolytic activity of immunoglobulin-depleted vs. non-depleted NHS. **(I)** Binding profile (IgG+IgA+IgM binding) of non-depleted NHS to transfected cells (blue: nuclear stain; red: AF647 anti-human IgG+IgA+IgM). **(J)** Binding profile (IgG+IgA+IgM binding) of immunoglobulin (Ig)-depleted NHS to transfected cells (blue: nuclear stain; red: AF647 anti-human IgG+IgA+IgM). **(K)** AChR decrease in AChR+Rapsyn-transfected cells incubated with tool anti-AChR antibody mAb35 in the presence of Ig-depleted NHS (n = 30) (One-way ANOVA (GraphPad Prism), Tukey’s multiple comparisons test). **(L)** Increased MAC deposition in AChR positive cells, incubated with tool anti-AChR antibody mAb35 in the presence of Ig-depleted NHS (n = 30) (One-way ANOVA (GraphPad Prism), Tukey’s multiple comparisons test). All NHS used and referred to in the remainder of the paper is Ig-depleted NHS. ****p <0.0001.

In order to make the case for a complement-targeted therapy in MG and identify patients appropriate for such a therapy, an assay system was sought that enabled stratification of MG patients according to the complement-activating ability of their anti-nicotinic AChR autoantibodies, differentiating them from MG patients with only ligand-blocking or crosslinking anti-AChR antibodies that do not activate complement and would not be amenable to complement therapies (**Figure 4A**). For this, a previously established CD46, CD55, CD59 triple knock out ARPE19 cell line (Gormley et al, manuscript in preparation) was transiently co-transfected with three plasmids, encoding for AChR subunits α1 & β1 (plasmid 1), δ & ϵ (plasmid 2) and the Rapsyn gene (plasmid 3) (**Figure 4B**). The presence of assembled AChR on the transfected cells was confirmed by staining with AF647-labelled α-Bungarotoxin. α-Bungarotoxin only stained cells co-transfected with plasmids 1 & 2, encoding for AChRα1β1 and AChRδϵ respectively (**Figure 4C**), or co-transfected with plasmids 1, 2 and 3, encoding for AChRα1β1, AChRδϵ and Rapsyn respectively (**Figure 4B**), but not cells which were transfected individually with either plasmid 1 (AChRα1β1) (**Figure 4D**) or plasmid 2 (AChRδϵ) (**Figure 4E**), or untransfected (**Figure 4F**). α-Bungarotoxin has two binding sites, which are between the α1 and δ and the α1 and ϵ subunits of AChR (20). Since the AChR pairs that provide the binding site for α-Bungarotoxin were on different plasmids, the results above confirm the expression of AChR subunits α1, δ, ϵ directly and of subunit β1 indirectly, in the double and triple transfected cells. AChR subunits α1, δ, ϵ were confirmed directly by α-Bungarotoxin binding. The β1 subunit was confirmed indirectly as it was on the same plasmid backbone and under the same SV40 promoter (**Figure S5**) as the ϵ subunit. The lack of binding of α-Bungarotoxin to cells, transfected with either AChRα1β1 only or cells transfected with AChRδϵ only, further confirms the specificity of the reagent to fully assembled AChR. As expected, the presence of the Rapsyn gene led to AChR clustering (**Figure 4B**), which was not the case for cells, transfected with all four AChR subunits α1β1δϵ, but without Rapsyn where the α-Bungarotoxin staining was homogenous (**Figure 4C**). Pooled serum from healthy donors depleted of immunoglobulins, used as a source of complement in the assay, had minimal residual IgG, IgA and IgM (**Figure 4G**), retained complement hemolytic activity albeit reduced (Ig-Depl NHS EC50 1.08% compared to NHS EC50 0.29%) (**Figure 4H**) and showed minimal non-specific binding to transfected cells, contrasting with the high non-specific immunoglobulin binding from NHS (**Figures 4I, J**). The triple-transfected cells were then incubated with the rat anti-human AChR tool antibody mAb35, washed and incubated with Ig-depleted NHS. AChR expression was detected using α-Bungarotoxin and MAC deposition using a C5b-9 antibody (**Figures 4K, L**). Compared to Ig-depleted NHS only, mAb35 in the presence of Ig-depleted NHS showed a significant decrease (p<0.0001) in AChR positive cells (**Figure 4K**) and a significant increase (p<0.0001) in MAC deposition within the AChR positive cell population (**Figure 4L**).

### MG patient autoantibodies display heterogenous binding to AChR on the cell surface

**Figure 5:**
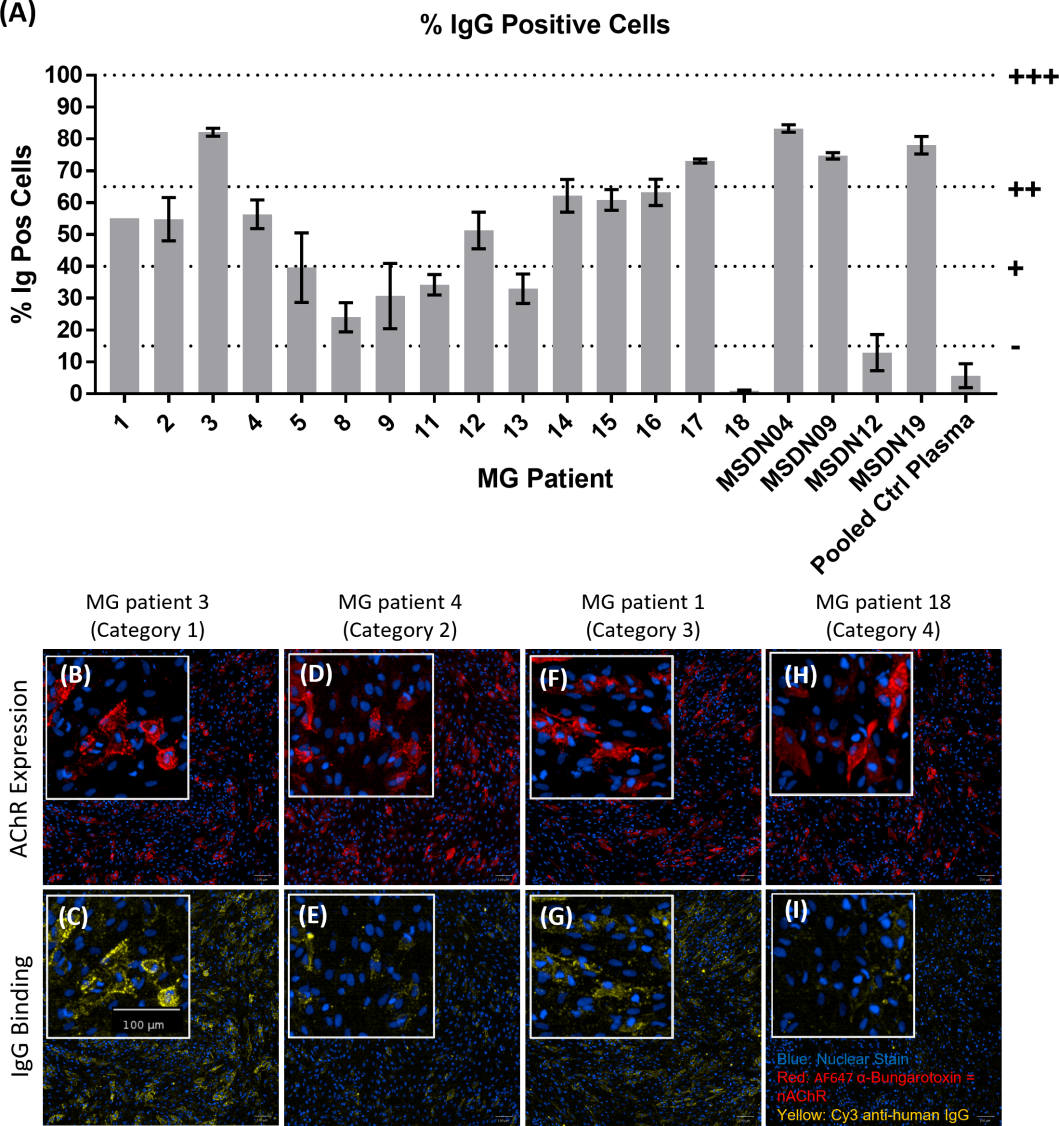
MG patient autoantibody binding pattern to AChR+Rapsyn transfected cells. **(A)** Image analysis summary graph, showing % IgG Positive cells. The bars represent the Mean ± SEM, n = 3 experiments, except for MG Patient 1 where n=1 experiment due to insufficient sample volume to perform additional repeats. Statistical significance was obtained using one-way ANOVA with Dunnet’s multiple comparison test, comparing the mean (n = 3 experiments) of each plasma sample to the mean of the pooled control plasma sample, except for MG Patient 1 (n = 1 experiment, 2 replicates), where statistical significance was obtained using an unpaired t-test, comparing the mean of the two MG patient 1 replicates to the mean of the two pooled control plasma replicates from the same experiment. **(B–I)** Representative images from each patient category showing AChR expression **(B, D, F, H)** and patient IgG binding **(C, E, G, I)**, with nuclear stain in blue, AF647 α-Bungarotoxin in red and Cy3 Anti-Human IgG in yellow. **(B, C)** MG Patient 3 (Category 1), **(D, E)** MG Patient 4 (Category2), **(F, G)** MG Patient 1 (Category 3), **(H, I)** MG Patient 18 (Category 4).

We screened MG patient plasma samples for IgG binding to the AChR+Rapsyn transfected cells; bound IgG was detected using Cy3-labelled anti-human IgG; AF647-labelled α-Bungarotoxin was used to stain the AChR clusters. **Figure 5A** summarizes IgG binding to the AChR positive cells corrected by subtracting background IgG binding to AChR negative cells. MG patients 3, 17, MSDN04, MSDN09, and MSDN19 showed strong binding to AChR - between 65% - 100% IgG positive cells (+++). MG patients 1, 2, 4, 12, 14, 15 and 16 showed moderate binding to AChR - between 40% - 65% IgG positive cells (++). MG patients 5, 8, 9, 11, and 13 showed low binding to AChR - between 15% - 40% IgG positive cells (+). MG patients 18 and MSDN12, as well as the pooled control plasma showed borderline to no binding to AChR - between 0% - 15% IgG positive cells (-). **Figures 5B-I** shows example images of cell staining with patient sera, representing different categories as summarized in **Table 1**. MG patient 3 (category 1) showed strong binding of IgG to the cell surface which followed the same binding pattern as α-Bungarotoxin (**Figures 5B, C**). MG patient 4 (category 2) and MG patient 1 (category 3) showed moderate binding of IgG to the cell surface in the same binding pattern as α-Bungarotoxin (**Figures 5D–G**). For MG patient 18, minimal background binding of IgG to the cell surface was visible, which was randomly spread, not following the same binding pattern as α-Bungarotoxin and was not picked-up by the analysis software algorithm (**Figures 5H, I**). All MG plasma samples, except for Patient 8, Patient 18, and patient MSDN12, showed statistically significant binding to the cells as compared to pooled control plasma (p-values ranging from 0.04 to <0.0001).

**Table 1:** Myasthenia Gravis patient categories according to how the patient Ig mediates AChR loss, MAC deposition and effect of C7 inhibition.

MG Patient	AChR Loss	AChR Loss blocked by anti-C7 Ab?	MAC Deposition	IgG Cell Binding	Anti-AChR Titre (nmol/L)	Category
2	**++**	**Yes**	**+**	**++**	5.43	1
3	**++**	**Yes**	**++**	**+++**	143.49	1
14	**++**	**Yes**	**+**	**++**	13.65	1
15	**++**	**Yes**	**+**	**++**	12.09	1
16	**++**	**Yes**	**+**	**++**	23.60	1
17	**++**	**Yes**	**++**	**+++**	67.52	1
MSDN04	**++**	**Yes (Partially)**	**+++**	**+++**	130.32	1
MSDN09	**++**	**Yes**	**+++**	**+++**	1.78	1
MSDN19	**++**	**Yes (Partially)**	**+++**	**+++**	53.84	1
4	**+**	**Yes**	**-**	**++**	4.80	2
12	**+**	**Yes**	**-**	**++**	10.13	2
13	**+**	**Yes**	**-**	**+**	3.80	2
1	**-**	**N/A**	**-**	**++**	18.40	3
5	**-**	**N/A**	**-**	**+**	16.28	4
8	**-**	**N/A**	**-**	**+**	2.18	4
9	**-**	**N/A**	**-**	**+**	3.21	4
11	**-**	**N/A**	**-**	**+**	4.37	4
18	**-**	**N/A**	**-**	**-**	41.89	4
MSDN12	**-**	**N/A**	**+**	**-**	<Detection Range	4
Pooled Ctrl Plasma	**-**	**N/A**	**-**	**-**	<Detection Range	N/A
Max Scale	**++**		**+++**	**+++**		

N/A, not applicable.

### C7 inhibition significantly reduced AChR loss mediated by complement-activating autoantibodies *in vitro*

**Figure 6:**
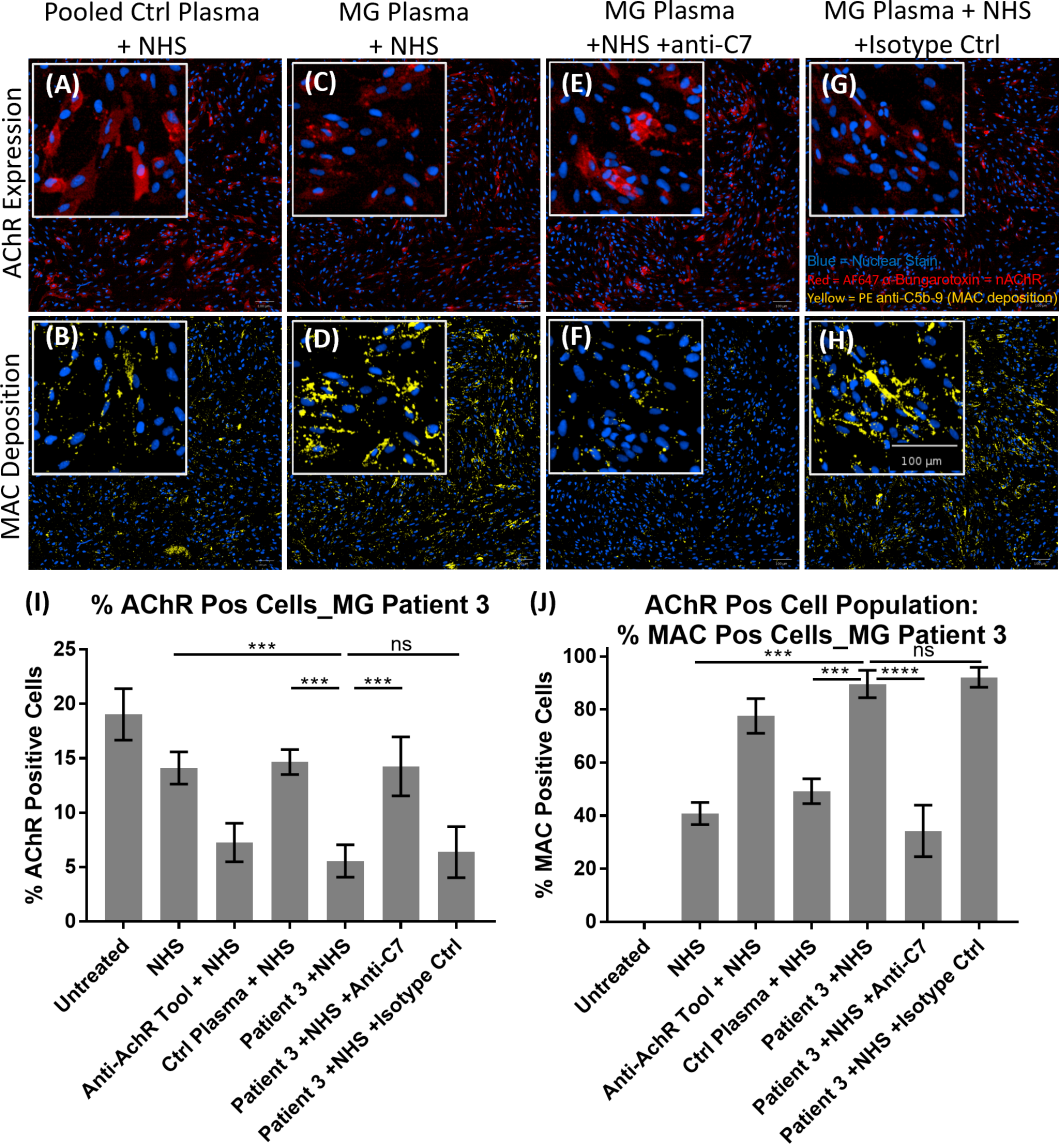
Example of MG patient plasma tested in the AChR loss and MAC deposition assay. **(A–H)** Representative images from MG patient 3 showing AChR expression **(A, C, E, G)** and MAC deposition **(B, D, F, H)**, with nuclear stain in blue, AF647 α-Bungarotoxin in red and PE anti-C5b-9 in yellow. “NHS” refers to Ig-depleted NHS in this figure. **(A, B)** Pooled control plasma + NHS, **(C, D)** MG Plasma + NHS, **(E, F)** MG Plasma + NHS +anti-C7, **(G, H)** MG Plasma + NHS +Isotype Ctrl. **(I, J)** Image analysis summary graphs for MG patient 3, showing % AChR positive cells **(I)** and % MAC positive cells within the AChR positive cell population **(J)**. The bars represent the Mean ± SEM, n = 3 experiments. Statistical significance was obtained using a repeated measures one-way ANOVA without correction, using Tukey’s multiple comparisons test. ***p <0.001, ****p <0.0001, ns, not significant.

The patient stratification assay was then validated with MG patient plasma as the source of anti-AChR autoantibodies (**Figure 6**). Representative images of AChR expression (**Figures 6A, C, E, F** **)** and MAC deposition (**Figures 6B, D, F, H** **)** show that, compared to pooled control plasma + NHS (**Figures 6A, B**), plasma from MG patient 3 + NHS (**Figures 6C, D**) showed a decrease in AChR staining (**Figure 6C** **)**, accompanied by an increase in MAC deposition (**Figure 6D** **)**. The presence of the anti-C7 antibody (TPP1820, referred to as “anti-C7 antibody”), in MG patient 3 plasma + NHS (**Figures 6E, F**) prevented the AChR loss and MAC deposition, but an isotype control antibody (**Figures 6G, H**) did not. The image analysis summary graphs (n=3 experiments) (**Figures 6I, J**) show that compared to NHS alone or pooled control plasma + NHS, plasma from MG patient 3 + NHS lead to a statistically significant decrease in % AChR positive cells with p = 0.0009 and 0.0006 respectively (**Figure 6I**), as well as a statistically significant increase in MAC deposition within the AChR positive cell population with p = 0.0002 and 0.0009 respectively (**Figure 6J**). Addition of the anti-C7 antibody completely prevented the AChR loss (**Figure 6I**) and MAC deposition (**Figure 6J**), whereas addition of an isotype control did not have any effect.

### Categorization of patient plasma according to complement dependent AChR loss

**Figure 7:**
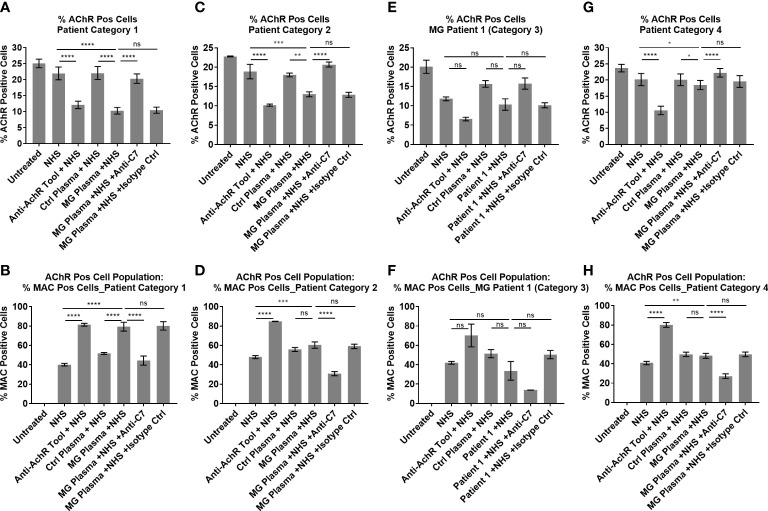
Patient Ig-mediated loss of AChR and MAC deposition - plotted means of Myasthenia Gravis patient categories, showing % AChR positive cells **(A, C, E, G)** and % MAC positive cells within the AChR positive cell population **(B, D, F, H)**. **(A, B)** Patient category 1, Mean ± SEM, n = 9 patients (mean of 3 experiments per patient, with two replicates each), **(C, D)** Patient category 2, Mean ± SEM, n = 3 patients (mean of 3 experiments per patient, with two replicates each), **(E, F)** Patient category 3, Mean ± SD, n = 2 replicates (1 patient, n = 1 experiment), **(G, H)** Patient category 4, Mean ± SEM, n = 7 patients (mean of 3 experiments per patient, with two replicates each). Statistical significance was obtained using a repeated measures one-way ANOVA without correction, using Tukey’s multiple comparisons test, except for category 3 where an ordinary ANOVA was used without pairing. *p <0.05, **p <0.01, ***p <0.001, ****p <0.0001, ns = not significant.

MG patient plasmas were grouped into four categories based on degree of IgG cell binding, ability to cause AChR loss and MAC deposition, and whether these can be blocked by the anti-C7 antibody (**Table 1**). The mean of all patient plasmas from each category was plotted and analyzed in GraphPad Prism (**Figure 7**). The in-house anti-AChR ELISA testing showed similar autoantibody titers as compared to the titers provided by the supplier (for samples where this information was available); however, there were discrepancies for some of the samples between autoantibody IgG binding to AChR on the transfected cells and ELISA antibody titers and therefore only the IgG cell binding data was taken into consideration when categorizing the patients as the patient autoantibodies would have bound to AChR on the cell surface, rather than plate-surface bound AChR.

Patient samples from category 1 showed high (++) AChR loss that was fully or partially blocked by the anti-C7 antibody, low (+) to high (+++) MAC deposition and moderate (++) to high (+++) IgG cell binding (**Table 1** and **Figures 5A–C**). When comparing NHS or pooled control plasma + NHS with MG plasma + NHS, patient samples from category 1 showed a statistically significant (p<0.0001) decrease in AChR expression (**Figure 7A**) and a statistically significant (p<0.0001) increase in MAC deposition (**Figure 7B**), which were blocked by the anti-C7 antibody, but not the isotype control antibody.

Patient samples from category 2 showed low (+) AChR loss that was fully blocked by the anti-C7 antibody, no detectable (-) MAC deposition and low (+) to moderate (++) IgG cell binding (**Table 1** and **Figures 5A, D, E**). MG plasma samples from category 2 + NHS showed a statistically significant decrease in AChR expression when compared to NHS alone (p=0.0003) or pooled control plasma + NHS (p=0.0014) (**Figure 7C**) and a statistically significant increase in MAC deposition when compared to NHS alone (p=0.0003), but not pooled control plasma + NHS (ns) (**Figure 7D**). The anti-C7 antibody, but not the isotype control antibody fully blocked the AChR loss (**Figure 7C**) and MAC deposition was blocked to below the detection level with NHS alone (**Figure 7D**).

Only one sample fitted into category 3; because of limited sample it was tested in a single experiment in duplicate. The profile comprised moderate (++) IgG binding to the AChR on the cells (**Table 1** and **Figures 5A, F, G**), but no significant AChR loss or MAC deposition, whereas the anti-AChR tool antibody did show a significant AChR loss (p=0.017) (**Figures 7E, F**, **Figure S10**). There was no information on disease score provided by the supplier for this sample.

Patient samples from category 4 showed no detectable (-) AChR loss, no MAC deposition and no (-) or low (+) IgG cell binding (**Table 1** and **Figures 5A, H, I**). MG plasma samples from category 4 + NHS did not show any biologically significant changes compared to NHS alone or control plasma + NHS for the AChR loss and MAC deposition readouts, but did show a statistically significant decrease in AChR expression (p=0.0333 when compared to NHS alone, p=0.0449 when compared to pooled control plasma + NHS) (**Figure 7G**) and MAC deposition (p=0.0097 when compared to NHS alone; not significant when compared to pooled control plasma + NHS) (**Figure 7H**). Inclusion of the anti-C7 antibody, but not the isotype control reduced MAC deposition to below the level seen with NHS alone (**Figure 7H**).

Statistically significant correlation was observed when comparing IgG cell binding vs. MAC deposition (r = 0.8, p < 0.0001 **Figure S14A** and **Table S4**), IgG cell binding vs. anti-AChR antibody titers (r = 0.5965, p = 0.007, **Figure S14B** and **Table S4**), anti-AChR antibody titers vs. MAC deposition (r = 0.4684, p = 0.0431, **Figure S14C** and **Table S4**).

## Discussion

The role of complement in human disease has been well described and many therapeutic concepts developed to target the system and harness the clear potential for therapeutic benefit in diverse diseases with significant unmet patient need (6, 21); however, few candidates have successfully progressed past Phase III clinical trials to approval. Two of the contributing factors are the choice of target and adequate patient stratification.

Therapies in the terminal pathway space have been focused on C5 (Eculizumab/Ravalizumab, Alexion Pharmaceuticals; Crovalimab, Roche; Zilucoplan, UCB) and, apart from a few recent examples, including C6 (Regenemab, CP010), other terminal pathway targets have received little attention (7, 8). The Eculizumab experience in PNH highlighted that not only the high target concentration and turnover, but also target-mediated drug disposition are contributing factors for the high dosing requirements. A mechanistic blind spot of C5 inhibition was highlighted with the identification of “C3 bypass” cleavage of C5 which allows C5 cleavage to occur in patients despite stoichiometrically adequate C5-inhibition (22). There are also variants of C5 that have been shown to have reduced or no binding to Eculizumab (23). Inhibition of the terminal pathway at a different point, such as C7, would overcome this and has additional advantages. Compared to C5, effective inhibition of C7 may be achieved at lower, less frequent doses *via* a subcutaneous route due to the lower plasma concentration and a likely more predictable, stable target concentration as C7 is not an acute phase reactant (24). Inhibition of C7 would also allow generation of C5a, which may have important safety implications as it would retain host defense and homeostatic functions of this fragment (25, 26).

In the present work, we report the characterization of three molecules from a discovery campaign that selected C7 inhibiting mAbs. In CP CH50 using normal human, cyno and rat sera, all three mAbs showed inhibition of human and cyno complement mediated lysis (**Figures 1A, B**) while TPP1657 and TPP1820 additionally inhibited rat serum (**Figure 1C**). The affinities of the mAbs for human and cyno C7 determined by SPR were mainly in the pM range (**Figure 1D**). In a previous report of four novel anti-C7 mAbs, reported affinities correlated with IC50 values in a comparable hemolysis assay (8). The two more potent mAbs from this study, with affinities of ~1nM, had comparable IC50 values to the mAbs in the present report where there was no apparent correlation between affinity and IC50 values. In terms of affinity and *in vitro* efficacy, the point of diminishing returns for C7 inhibition may therefore lie at affinities of ~1nM with further differences in efficacy driven by each mAb’s unique mechanism of action.

We used HDX-MS to map the C7 epitopes of the mAbs (**Figure 1E**) and further elucidated the mechanism of action for each mAb using a BLI assay to model the stepwise assembly of the MAC (**Figure 2A**). Three distinct epitopes, with different mechanisms of action were identified. TPP1820 bound the FIM domains (**Figure 1E**) and prevented binding of C7 to C5b6 in the BLI assay (**Figure 2B**). The C7 FIM domains bind the C345C domain of C5 in the C5b6 complex, a critical step in MAC formation (27–29). TPP1653 bound the pore-forming MACPF domain (**Figure 1F**); mapping of the HDX protection patterns on the MAC structure (**Figure 1G**) suggested that the transition of region 335-362 from disordered loop to structured β-sheet, and its move away from the 393-408 pattern, were the critical steps in inhibition of MAC formation by TPP1653. In the BLI assay, TPP1653 partially inhibited binding of C7 to C5b6, but not C8 binding to C5b7 (**Figure 2C**). These data support the mechanism suggested by the HDX-MS data (**Figure 1G**) as the β-hairpin is only unfolded after binding to C5b6, rendering the C5b7 complex lipophilic (30, 31). This means that TPP1653 likely prevents the critical initial anchoring of C5b7 to the cell membrane, thus preventing formation of MAC but not the soluble TCC. TPP1657 binds an epitope on the LDLRA domain (**Figure 1H**), possibly bridging the bottom of the MAC pore-forming domain; the BLI assay showed that TPP1657 inhibited C8 binding to C5b7 (**Figure 2D**). This is a novel finding as the importance of the C7 LDLRA domain for C8 binding had not been shown previously. These data provide the first description of functionally relevant epitopes on C7 that are tractable for inhibition.

TPP1820 was selected for an *in vivo* study due to the better potency in the rat hemolysis assay. (**Figure 1C**). Passive experimental MG (EAMG) in rats, induced by injection of a complement-fixing anti-AChR mAb (mAb35), is a well-established model replicating acute clinical features of the human disease (32, 33). TPP1820, given prophylactically at the time of disease induction or therapeutically 16h post-disease induction, significantly suppressed clinical disease in the EAMG model in comparison to isotype control treated animals when assessed at 24, 30 and 48 hr (**Figures 3A, B**). These findings are in line with previous reports of C5 and C7 inhibition in this model (8, 34, 35). As the data presented are derived from a single, high-dose study it is not possible to draw any comparative conclusions on the efficacy of TPP1820 versus other mAbs that were previously assessed in this model. Nonetheless, these data show that C7 inhibition, prophylactically or therapeutically during acute pathology, protects rats from developing experimental MG and provides further validation of C7 as a target in MG.

MG is a heterogenous disease, both at the level of auto-antigen (AChR, muscle-specific kinase (MuSK), LDL receptor related protein 4 (LRP4/agrin)) and within each serological subtype (36). For anti-AChR positive MG, this has been highlighted in the Eculizumab REGAIN study which failed to show a statistically significant difference between Eculizumab and placebo (13). To address the issue of patient stratification and facilitate future trial design, we developed a cell-based patient stratification assay and undertook a proof of concept study in small cohort of patients (n=19). The assay reproduced MG disease biology *in vitro* and showed that 12 out of 19 patients (patient categories 1 + 2) had clear C7-dependent loss of AChRs (**Table 1**), identifying a subset of patients with pronounced complement involvement and therefore likely to benefit from terminal pathway complement therapy. Potential alternative mechanisms in the other seven patients were not evaluated due to heterogeneity and limited samples. Patient IgG binding and MAC deposition showed a stronger correlation than autoantibody titers and MAC deposition, suggesting that cell binding is a more meaningful measure than the antibody titre (**Figure S14**). Patient IgG binding also correlated with autoantibody titers, however two obvious outliers were observed in the small patient cohort tested here. A recent study by Obaid et al. showed a similar level of heterogeneity of MG patient Ig ability to cause MAC formation in a larger cohort and similar trend to correlation of autoantibody cell binding and MAC deposition (37). While we applied our assay to a smaller cohort, we titrated the levels of normal NHS to sub-lytic levels to prevent lysis of cells impacting quantification of MAC deposition and AChR loss which may result in higher sensitivity in our system. This assay provides a useful tool for patient stratification and as well as identifying the most relevant patients for MAC-targeting therapies but is also applicable to testing of inhibitors upstream of MAC, e.g. C1q and C3.

However, the complexity of the cell-based assay we developed presents challenges for its use in a clinical setting in the present form. A flow cytometry assay using stably transfected cells and detecting C3/MAC deposition may be more practical. Here, the assay system developed by Obaid et al, is more suited. The complement regulator deficient ARPE-19 were chosen as tool to interrogate the MAC-dependent mechanisms and the ability to perform reproducible transfection, similar to the choice of HEK cells by Obaid et al, 2022 (37). If assay throughput was less of a requirement, primary cells or a myoblast cell line, would be preferred over an epithelial line for physiological relevance. Plomp et al, used murine hemi-diaphragms to model the complement activation using a tool anti-AChR mAb and the downstream functional effects, but the setup is less well suited to a clinical setting (38). It is important to emphasize that we are unable to assess the predictive value of this *in vitro* system and verify the exact disease mechanism in patients. For this, the assay would require validation using e.g. historical patient samples that have received anti-complement or other therapies with associated response/non-response data. Inclusion of a cohort of patient samples with an unrelated autoimmune disease would confirm specificity of our assay beyond the healthy donor plasma included here.

In conclusion, we characterized a set of novel anti-C7 monoclonal antibodies, and provided novel insights into tractable, functionally relevant epitopes on C7 and further validation of C7 as target for MG. With view to improved clinical trial design, we report a proof of concept patient stratification assay, developed to assess the heterogeneity of complement-dependence in MG. Taken together, these findings are relevant to future development and testing of new complement therapies in MG and other terminal pathway mediated pathologies, to facilitate bringing the right drug to the right patient with the associated benefits of faster and better treatment outcomes for patients and lowered burden on healthcare systems for society.

## Data availability statement

The raw data supporting the conclusions of this article will be made available by the authors, without undue reservation. Anti-C7 mAbs can be made available under MTA to repeat the in vitro experiments. The CRISPR edited APRE-19 cell line cannot not be shared due to licensing limitations. A protocol to replicate the effect of the regulator k/o is included as supplementary file.

## Ethics statement

Ethical review and approval was not required for the study on human participants in accordance with the local legislation and institutional requirements. The patients/participants provided their written informed consent to participate in this study. All animal experiments were approved by the Committee for Animal Care, Welfare and Use committee. All animal studies were ethically reviewed and carried out in accordance with European Directive 2010/63/EEC and the GSK Policy on the Care, Welfare and Treatment of Animals.

## Author contributions

E-MN, SD, BM, MF, TW, and SK contributed to design of the study and supervised. EL, WZ, DGow, CS, IO, LS, DGorm, AS, AB, EW, RP, MB, and SP-F performed experiments, analysis, and statistical analysis. MF and E-MN performed review of data integrity. EL and E-MN wrote the first draft of the study. All authors contributed to the article and approved the submitted version.

## Funding

BM and WZ would like acknowledge UK Dementia Research Institute and Alzheimer's Research Race Against Dementia Fellowship.

## Acknowledgments

BM is supported by the UK Dementia Research Institute (UK-DRI), funded in part by the Medical Research Council. WZ is a Race Against Dementia Fellow and UK-DRI Future Leader Fellow. The authors would like to acknowledge the expert contributions of Joselli Silva O’Hare, Victoria Martin and Irene Sanjuan-Nandin in the *in vivo* campaign and selections.

## Conflict of interest

EL, DGow, CS, IO, LS, DGorm, AS, AB, EW, MB, TW, RP, SK, MF, SD, E-MN are employees and shareholders of GSK. SP-F is an employee of GSK.

The remaining authors declare that the research was conducted in the absence of any commercial or financial relationships that could be construed as a potential conflict of interest.

## Publisher’s note

All claims expressed in this article are solely those of the authors and do not necessarily represent those of their affiliated organizations, or those of the publisher, the editors and the reviewers. Any product that may be evaluated in this article, or claim that may be made by its manufacturer, is not guaranteed or endorsed by the publisher.

## Supplementary material

Supplementary File 1 Containing supplementary data and methods for the HDX-MS and BLI experiments:

Supplementary File 2 Generation of complement regulator deficient ARPE-19 cells and protocol for inhibition of complement regulators in lieu of the cell line.

Supplementary File 3 Containing supplementary data for the MG patient stratification assay.

Supplementary Figure 5 Plasmid maps of pBiCIH-hnAChRα1β1 and pBiCIN-hnAChRδϵ, created in SnapGene 5.3.2.

Supplementary Figure 6 Plasmid map of PCIP4_Rapsyn, created in SnapGene 5.3.2.
