# Supplementary material for: Discovery of functionally distinct anti-C7 monoclonal antibodies and stratification of anti-nicotinic AChR positive Myasthenia Gravis patients
Source: Front Immunol. 2022 Sep 5;13:968206. doi: 10.3389/fimmu.2022.968206 (PMC9486540; doi:10.3389/fimmu.2022.968206)
Supplement: Supplementary File 1 — Containing supplementary data and methods for the HDX-MS and BLI experiments: [file DataSheet_1.pdf]

Figures & Tables for supplementary Section

Figures:
Figure S1: Sequence coverage and redundancy
Figure S2: Differential fractional uptake and time course data vs. peptide ID
Figure S3: Woods plot
Figure S4: Volcano plot analysis

Tables:
Table S1: HDX Experimental Data Summary
Table S2: Table of peptides with statistically significant protection

Experimental details and parameters

Figure S1: Sequence coverage and redundancy

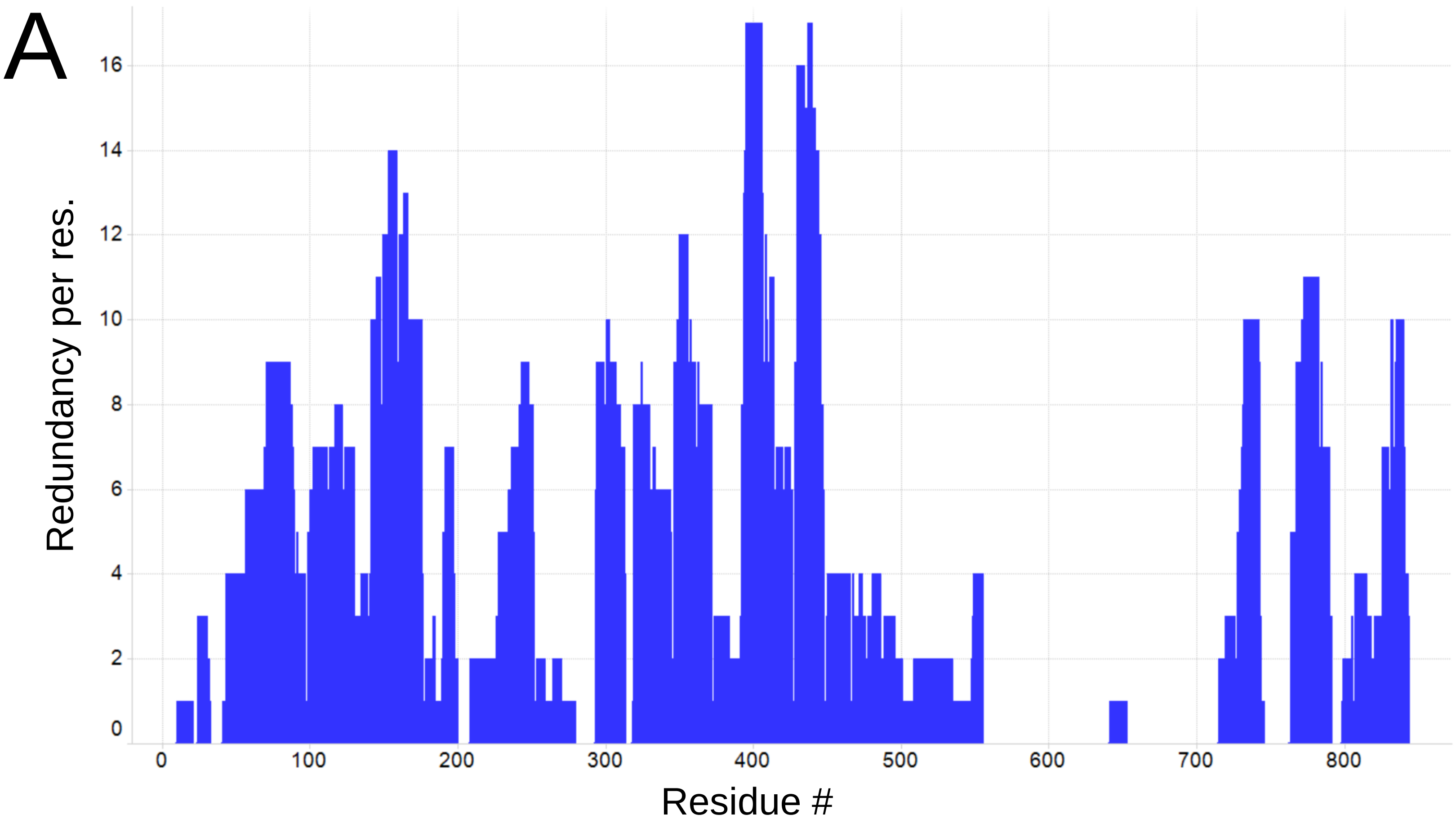

Figure S2: Differential fractional uptake and time course data vs. peptide ID

B

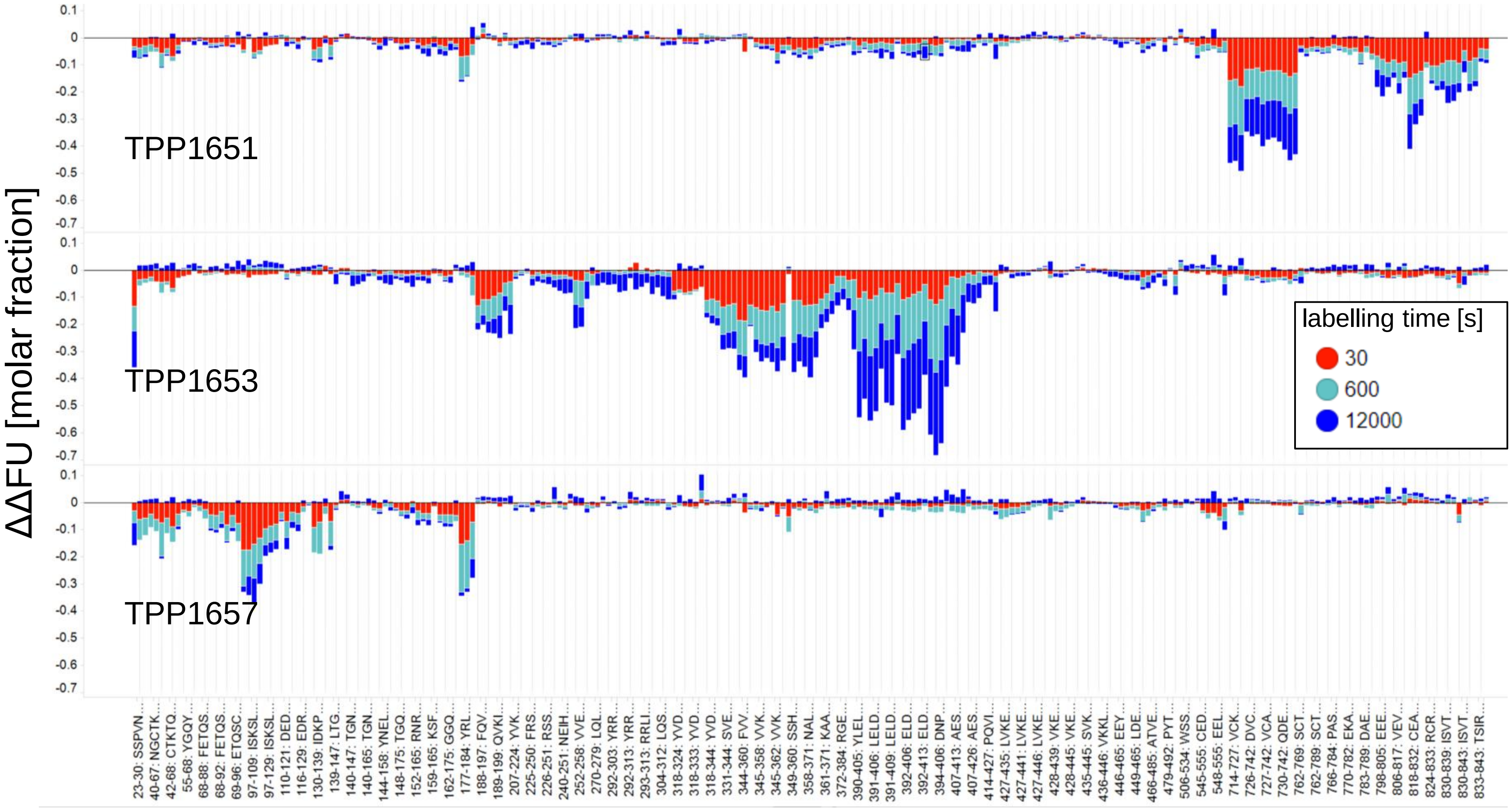

Figure S3: Woods Plot

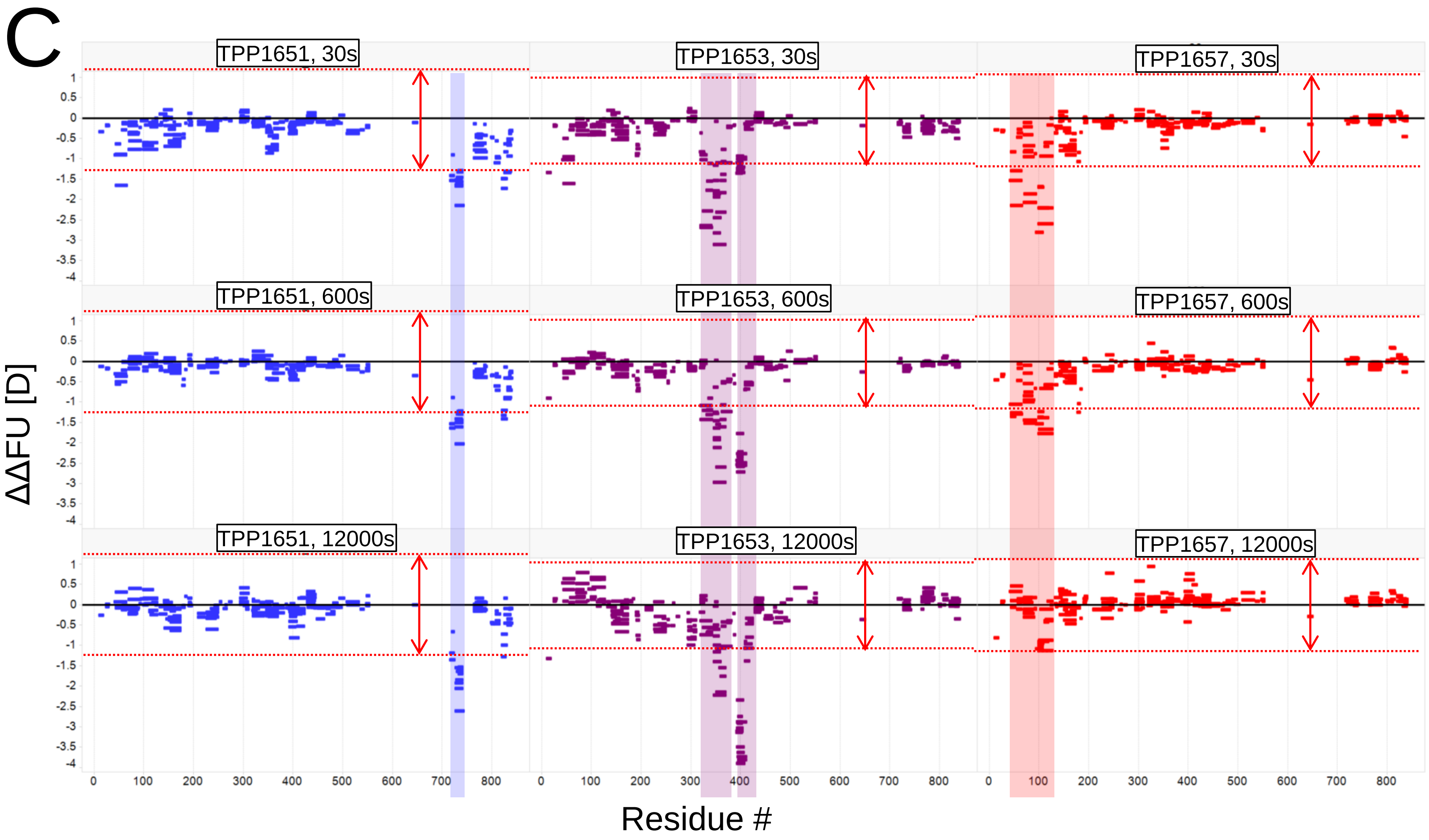

Figure S4: Volcano plot analysis, p=0.01

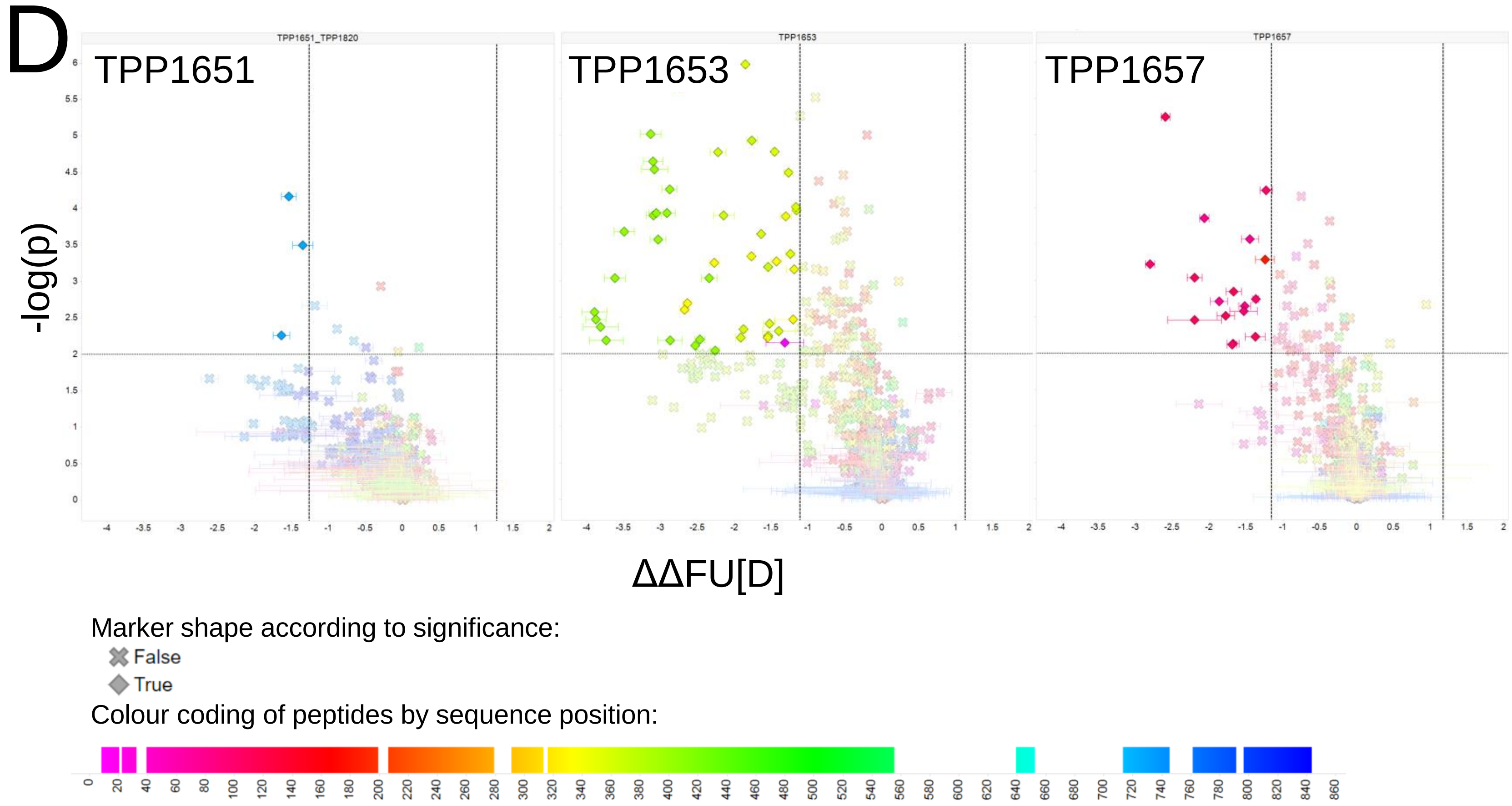

filename: ELN43007_1_HDX_DB_viewer.dxp

Figure S1-4 legends

S1 Peptide map of C7: coverage and redundancy;

S2 Plot of differential fractional uptake values (ΔΔFU) of TPP1651 (top), TPP1653 (middle) and TPP1657 (bottom) in complex with C7, expressed as molar fraction of the maximum theoretical uptake for each peptide and normalised for the length of peptide;

S3 Associated Woods plots of the three complexes for separated by labelling time. The dotted lines indicate significance threshold at $p<0.01$. Regions that met the significance criteria (see Table HDX1) were highlighted.

S4 Volcano plot analysis of the three complexes. Peptides that represent significant hits at $n=3$, $p<0.01$ are highlighted and indicated by diamonds (see Table HDX2. Peptides below the threshold are indicated by crosses. The colour coding shows the position of the peptide in the sequence.

Table S1: Summary of Experimental Data

**Protein States**	Apo-C7, complexes of C7 with TPP1651, 1653 and 1657
**Instrument Details**	Liquid Handling: Leap2 Robot (Leap Trajan)<br>UPLC-MS: Acquity HDX platform with SynaptG2Si mass spectrometer (Waters)
**Sample details**	Peptide mapping: 30pmol C7 in 5ul sample buffer;<br>Labelling time course: 15pmol C7 in 5ul sample buffer as apo reference. Complexes were prepared by addition of 10pmol mAb (=0.7x molar excess).
**Reaction details**	H2O sample buffer: 50mM MOPS, 150mM NaCl in H2O, pH7.2;<br>D2O labelling buffer: 50mM MOPS, 150mM NaCl in D2O, pD6.8 uncorrected (theoretical maximum incorporation= 91%D);<br>labelling temperature 20degC;<br>labelling times: 30, 600, 12000s<br>quench buffer: 6M urea, 1M TCEP, 400mM formate, pH3.5<br>quench, trapping and LCMS temperature 0degC;<br>quench time: 15min<br>trapping time: 4min<br>Pepsin digestion (Enzymate, Waters) at 15degC.
**Data acquisition**	solventA: 0.2% formic acid +0.03% TFA; solventB: 0.2% formic acid in acetonitrile<br>analytical gradient: 11-28% solventB in 10min, then 28-40% in 2min.<br>MS data were recorded with ion mobility, i.e. in HDMSE mode for peptide mapping and HDMS mode for labelled samples.
**Coverage**	249 peptides, 75.7% coverage, average length 15.4 aa.
**Significance threshold for differential HDX**	Volcano Plot analysis: Statistical significance of differential HDX was tested at p=0.01 confidence level. HDX protection was considered significant if ≥ 3 peptides, or time points of the same peptide, in any contiguous region of the C7 sequence exceeded the significance threshold (see Woods plot). Significant thresholds and residue ranges with significant ΔΔFU were:<br>TPP1651 complex: ±1.27D, res. 714-727;<br>TPP1653 complex: ±1.12D, res. 318-371 and 390-413;<br>TPP1657 complex: ±1.16D, res. 68-129.<br>Assuming 13.4 exchangeable D for a peptide of average length this corresponded to a ΔΔFU Threshold of ca. 9%;
**HDX data**	A table of peptides and time points with statistically significant ΔΔFU is attached.

Table S2: List of C7 peptides experiencing statistically significant protection P<0.01

VolcanoPlotP0.01

3.) General supporting Information

C6-C7 Residue Concordance of HDX Volcano Plot Hits

Clone (TPP)	SPR Bin	C7 HDX construct	C6 (crystal structure 3T5O)	ELN Reference
TPP1651&1820	1	714-727	765-779	N70158-1-06 & N70158-7-04
TPP1653	3	318-371, 390-413	359-417, 436-458	N70158-1-06
TPP1657	2	68-129	101-160	N70158-1-06

sequence alignment produced with DNAStar MegalignPro 17.1.1
filename: C6C7_alignments.msa

Experimental Parameters

- Experiment date: Oct 26, 2018
- Samples:
  C7 (Complement Technologies #A124, lot 11b)
  TPP-1651 (N61099-57-8; protection pattern identical to TPP1820, as shown in N70158-7-04)
  TPP-1653 (N61099-57-2)
  TPP-1657 (N61099-57-5)
- Main Experimental Parameters:
  C7 concentration: 30pmol for peptide mapping, 15pmol for labelling;
  0.7eq mAb for complex samples;
  labelling buffer: MOPS pD6.8 uncorrected;
  labelling time: 4 time points, 0, 30, 600 and 12000s @20deg;
  replication: 3 replicates
  blanks: before and after blanks to assess background and carryover;
  quench buffer: 6M Urea +1.5M TCEP, pH3.5;
  quench time: 15min@0deg for labelling.
  Digestion column: Waters Enzymate (pepsin)
- Processing & analysis:
  PLGS &HDExaminer.
  Peptides with poor s/n, high background in blank and unidentified 100% signal were eliminated.
  Heatmap data were calculated with "very heavy" smoothing.

Output

- Output:
  Volcano plot, differential fractional uptake and average heatmap data.

- File names:
  Original HDExaminer file with all the mAbs: 70158_1_06_c02.hdx
  File with 1651=1820, 1653, 1657: ELN43007_1.hdx
  states file: ELN43007_1_statestable.csv
  HDX database entry #1722

C6crystal/C7HDX Global Alignment

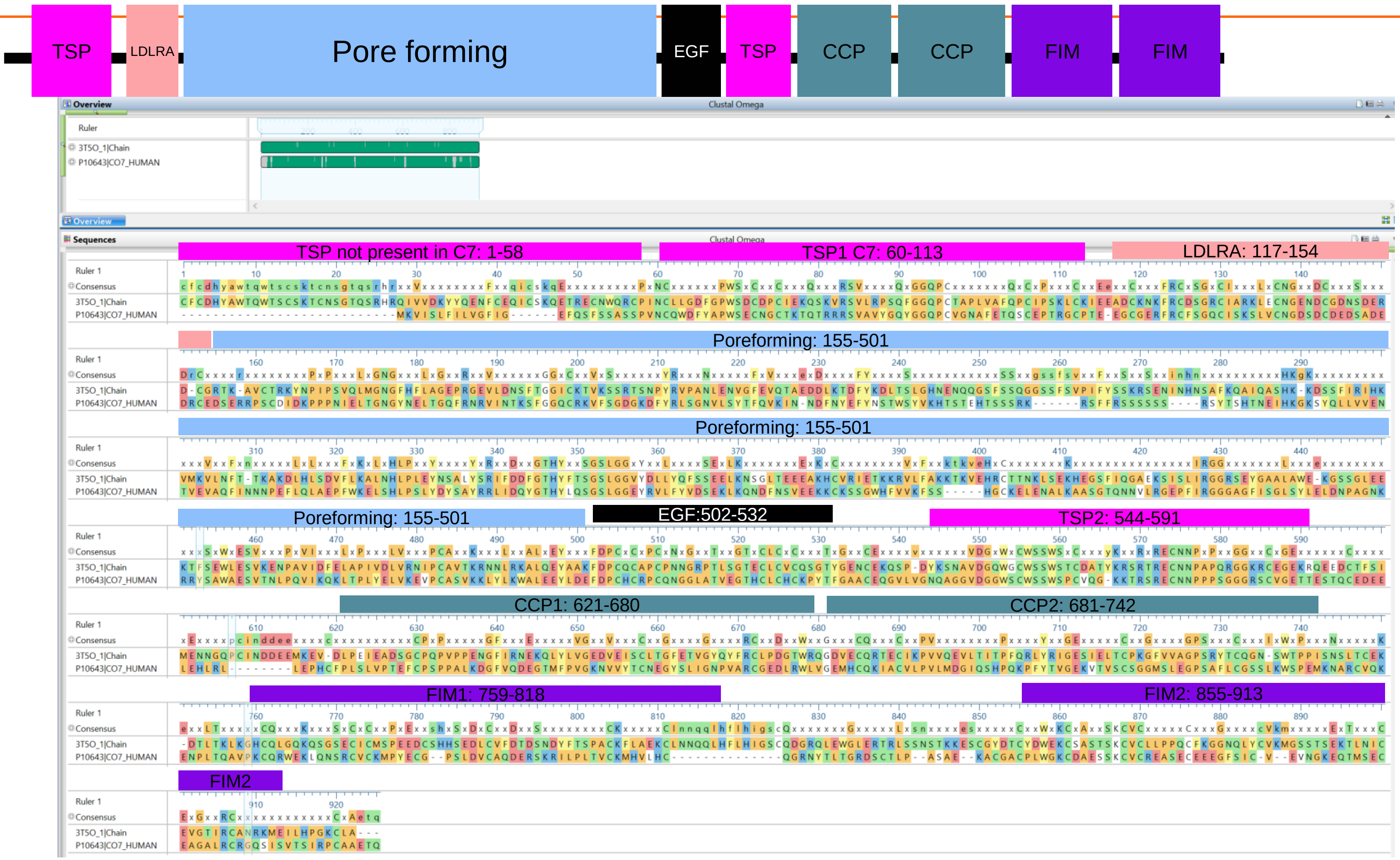

C6 sequence and domain information from: https://www.rcsb.org/sequence/3T5O
C7 sequence: https://www.uniprot.org/uniprot/P10643
sequence alignment produced with DNAStar MegalignPro 17.1.1
filename: C6C7_alignments.msa
