# Supplementary material for: Discovery of functionally distinct anti-C7 monoclonal antibodies and stratification of anti-nicotinic AChR positive Myasthenia Gravis patients
Source: Front Immunol. 2022 Sep 5;13:968206. doi: 10.3389/fimmu.2022.968206 (PMC9486540; doi:10.3389/fimmu.2022.968206)
Supplement: Supplementary File 2 — Generation of complement regulator deficient ARPE-19 cells and protocol for inhibition of complement regulators in lieu of the cell line. [file DataSheet_2.pdf]

Cell Line Generation

CD46, CD55 and CD59 target specific CRIPSR/Cas9 *E. coli* expression plasmids were generated using the GeneArt® CRISPR Nuclease Vector with CD4 Enrichment Kit (ThermoFisher Scientific, Loughborough, UK) and undertaken in accordance with the manufacture's protocol and plasmids were isolated using QIAprep Spin Miniprep Kit (Qiagen, Hilden, Germany). Plasmid DNA was sequenced by Sanger sequencing on the ABI 3730xl DNA Analyser (Applied Biosystems) to ensure the correct target sequence was successfully transformed. Sequence alignment and analysis was undertaken using MegAlign Pro, part of the Lasergene Core Suit 12 package (DNAStar, Madison, WI, USA) and plasmids with the correct gRNA insert were selected for a larger scale expression. The plasmids were then isolated and purified at a high concentration using the PureLink Hi Pure Plasmid Filter Purification Kit

CD46	Top *In-silico* oligo	5' CAATTGTGTCGCTGCCATCG 3'
	DNA Strand targeted	Reverse
	Top strand oligo	CAATTGTGTCGCTGCCATCG
	Top stand oligo with **overhangs**	CAATTGTGTCGCTGCCATCG**GTTTT**
	Bottom stand olgio	CGATGGCAGCGACACAATTG
	Bottom stand oligo with **overhangs**	CGATGGCAGCGACACAATTG**CGGTG**
CD55	Top *In-silico* oligo	5' TCACTGAGTCCTTCTCGCC 3'
	DNA Strand targeted	Forward
	Top strand oligo	TCACTGAGTCCTTCTCGCC
	Top stand oligo with **overhangs**	TCACTGAGTCCTTCTCGCC**GTTTT**
	Bottom stand olgio	GGCGAGAAGGACTCAGTGA
	Bottom stand oligo with **overhangs**	GGCGAGAAGGACTCAGTGA**CGGTG**
CD59	Top *In-silico* oligo	5' ACGACGTCACAACCCGCTTG 3'
	DNA Strand targeted	Forward
	Top strand oligo	ACGACGTCACAACCCGCTTG
	Top stand oligo with **overhangs**	ACGACGTCACAACCCGCTTG**GTTTT**
	Bottom stand olgio	CAAGCGGGTTGTGACGTCGT
	Bottom stand oligo with **overhangs**	CAAGCGGGTTGTGACGTCGT**CGGTG**

(Invitrogen™) according to the manufacturer's protocol.

Table 1; Forward and reverse RNA oligo sequences identified in silico to minimise off target effects. For each gene target the complementary forward and reverse oligo with the required overhang sequence for vector ligation.

Wild type ARPE-19 cells were cultured as previously described and seeded into required number of T75 flasks at 1 x10^6 cells per flask and left to adhere overnight. Transfection reagents were scaled up and the cells were transfected with 19µg of CRISPR plasmid containing the CD46, CD55 or CD59 gRNA

insert using Lipofectamine™ 3000 following manufacture's protocol. Media was changed after 24 hours. $1x10^6$ cells from each transfection condition were analysed by flow cytometry on a FACS Canto II (Beckman Dickenson® Bioscience, Oxford, England), to confirm loss of complement regulators by staining with 1µg/ml APC-conjugated anti-human CD46 (clone TRA-2-10, Biolegend), 1µg/ml PE-conjugated anti-human CD55 (clone JS1, Biolegend) 1µg/ml or FITC-conjugated anti-human CD59 (Clone p282, Biolegend) for one hour, on ice, in the dark and after washing, stained with 1:100 LIVE/DEAD® Fixable Violet Dead Cell Stain. All samples were gated on single, live cells, and analysis was carried out on greater than 10,000 events using FlowJo V10 (FlowJo, Ashland, OR, USA).

Cells transfected with the CD46, CD55 or CD59 plasmid were expanded and depleted of regulator expressing cells by taking 1 $x10^7$ cells per condition and adding 5µg/ml anti-CD46 (Hycult, HM2103), 5µg/ml of anti-CD55 (Clone 216, BRIC) and 5µg/ml of anti-CD59 (Clone 229, BRIC). Cells were incubated in the antibody solution on ice for 1 hour, washed and resuspended in 80µL of MACs buffer. Cells were then magnetically labelled using 20µL of anti-mouse IgG microbeads (Miltenyi Biotec), mixed well and incubated on ice for 15 minutes, washed and resuspended in 500µL of MACs buffer. LD MACs columns (Miltenyi Biotec) were prepared and equilibrated in accordance with manufacture's protocol. Once equilibrated, transfected cell population was added to an individual column, allowed to enter the column, and washed with 3x 3ml of MACs buffer. The flow through, containing the negative population, were collected before the column was removed from the magnet and the retained cell population flushed off the column with 5ml of MACs buffer and collected separately. Both positive and negative cell populations were centrifuged at 200xg for 5 minutes, resuspended in culture media and returned to separate flasks for culture for further downstream analysis and validation.

Cell Line Validation

Cultured cells were lysed in RIPA buffer (Pierce, Waltham, MA, USA) containing protease and phosphatase inhibitor cocktail (ThermoFisher) at a density of 1 x10^6 cells per 30µL lysis buffer then sonicated for ~1 minute using the Diagenode BIORUPTER (ThermoFisher). Cell debris was pelleted using an ultra-centrifuge (Optima™ TLX, Beckman Coulter) at 100,000rpm for 30 minutes at 4°C. Leaving the pellet undisturbed, the soluble fractions of the cell lysates were removed, transferred into clean Eppendorf tubes and stored at -20°C until required.

Cell lysate proteins were resolved via SDS-PAGE and transferred to nitrocellulose membranes (ThemoFisher) using the TE22 Mighty Small transfer tank (Hoefer, Holliston, USA). Once transfer was complete, the membranes were blocked with Odyssey blocking buffer (LI-COR, Cambridge, England) for ~3 hours followed by incubation with regulator specific primary antibodies, 1µg/ml anti-CD46 (Hycult, HM2103), 1µg/ml of anti-CD55 (Clone 216, BRIC), 1µg/ml of anti-CD59 (Clone 229, BRIC) and loading control rabbit-anti-histone-H3 mAb (clone 17H2L9**,** Thermofisher) with gently agitation overnight at 4°C. Membranes were washed four times with MQ PBS + 0.1% Tween^{20}. IRDye® 680RD Goat Anti-Mouse IgG (Licor) and IRDye 800CW Donkey Anti-Rabbit IgG (Licor) secondary antibodies were diluted 1:15,000 in 10ml/membrane of 1x Odyssey blocking buffer then left to incubate for 1 hour wrapped in foil to protect from light. Membranes were washed as previously described and air dried before imaging on the Odyssey CLx imager (LI-COR).

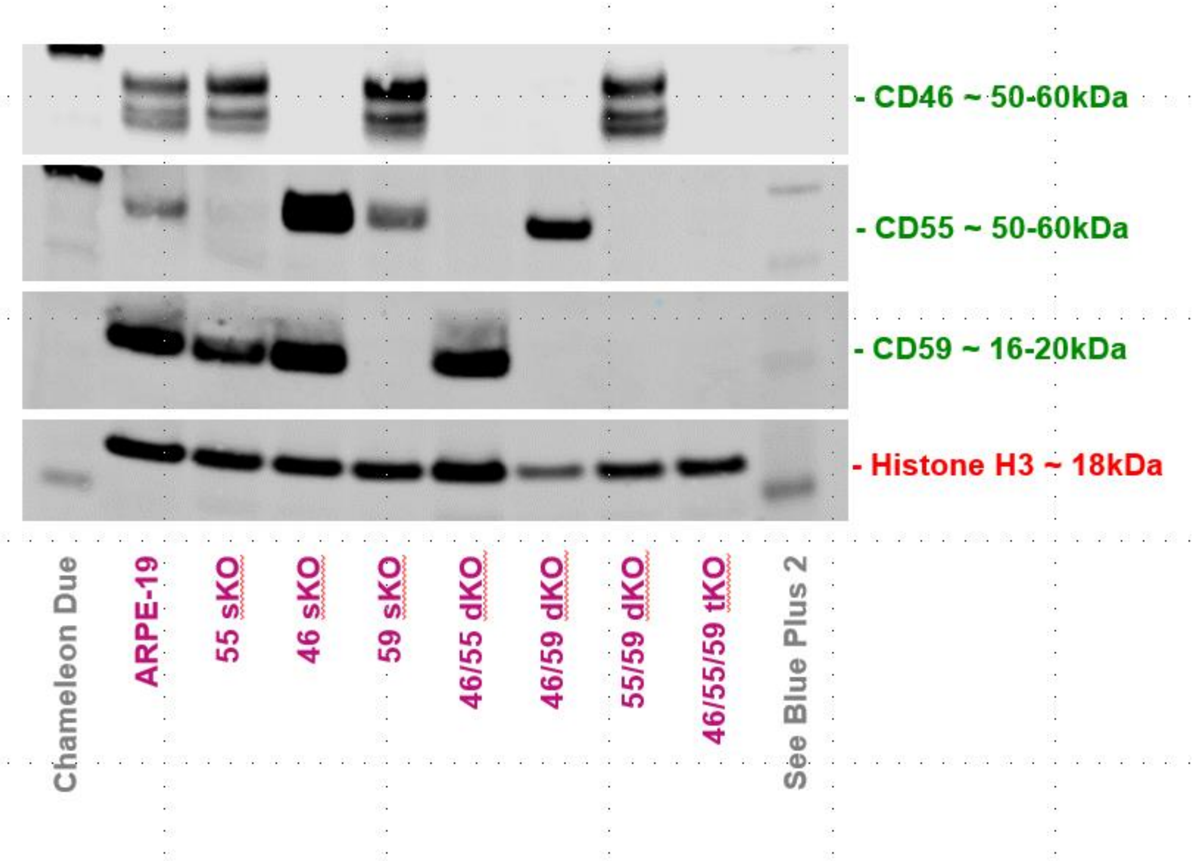

Composite of 3 Odyssey western blots of ARPE-19 WT and the complete range of complement regulator single, double and triple KO cell lysates probed with mouse-anti-human CD46, CD55 or CD59 primary mAb and detected with IRDye® 680RD Goat Anti-Mouse Ig to determine complement regulator protein expression and rabbit anti-human Histone H3 rab-mAb detected with IRDye 800CW Donkey Anti-Rabbit IgG loading control.

Protocol to block complement regulators in lieu of using a k/o cell line

There are many different cell types that would be suited for use in the patient stratification assay. Most cells express complement regulators and for effective MAC deposition and lysis, CD55 and CD59 can be blocked by using anti-CD55 (Clone 216, BRIC) and anti-CD59 (Clone 229, BRIC)). The mAbs are validated functional inhibitors and are commercially available. Working concentration should be established for each cell type and experimental conditions by titration of each mAb.
