# Supplementary material for: Discovery of functionally distinct anti-C7 monoclonal antibodies and stratification of anti-nicotinic AChR positive Myasthenia Gravis patients
Source: Front Immunol. 2022 Sep 5;13:968206. doi: 10.3389/fimmu.2022.968206 (PMC9486540; doi:10.3389/fimmu.2022.968206)
Supplement: Supplementary File 3 — Containing supplementary data for the MG patient stratification assay. [file DataSheet_3.pdf]

Supplementary File 3

S5: Plasmid maps – AChR Subunits

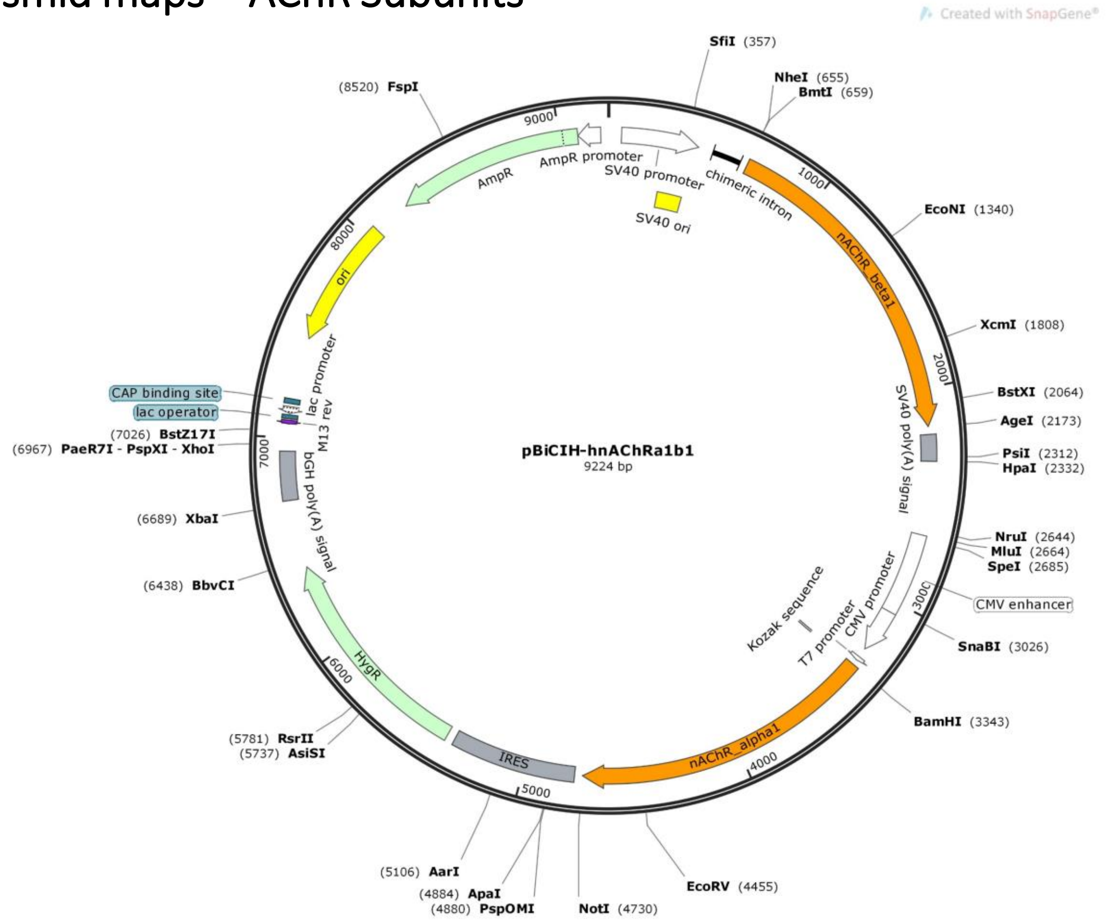

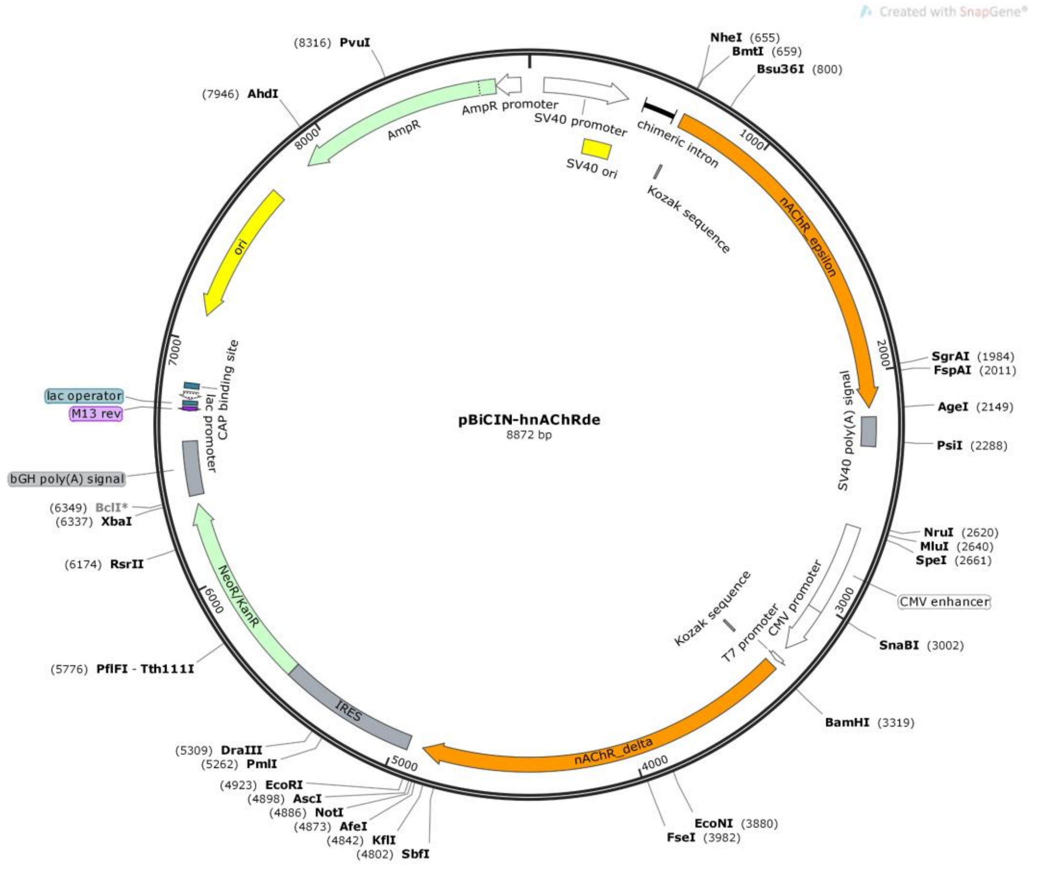

S6: Plasmid map – Rapsyn

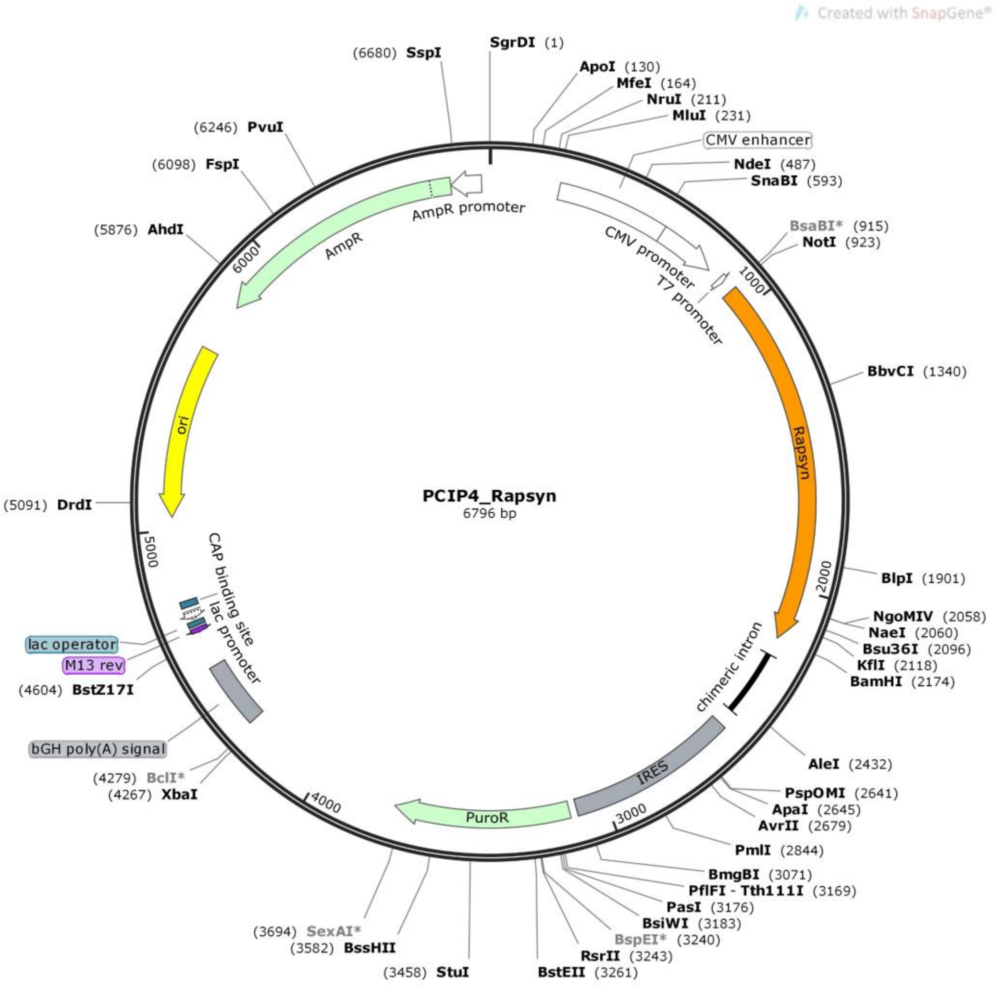

S7: Additional patients – AChR binding

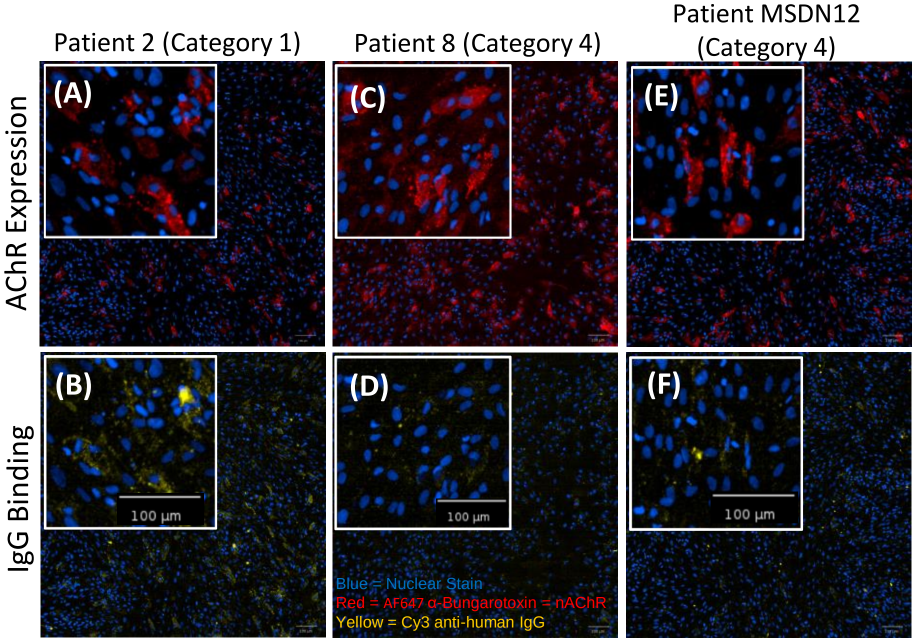

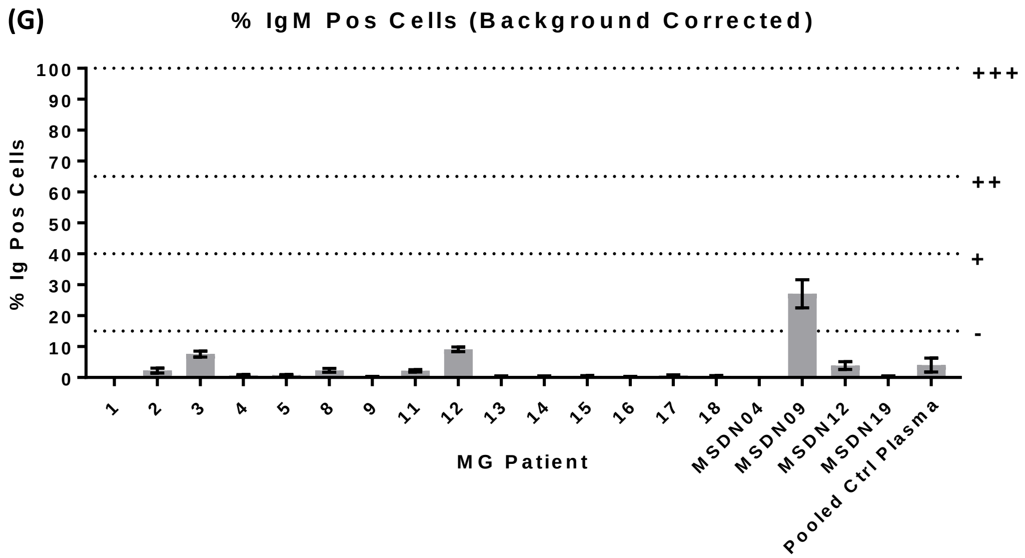

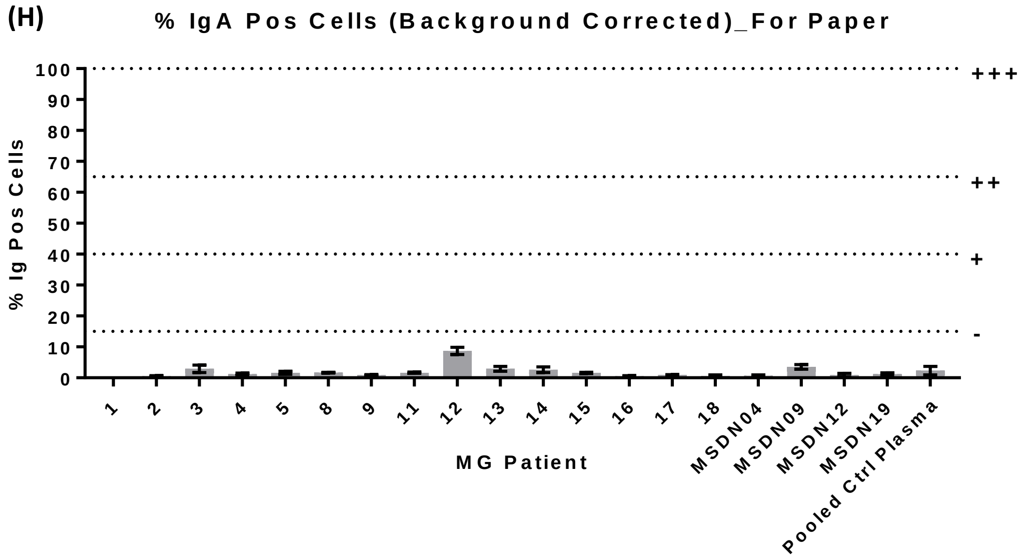

S7: Examples of additional patient sample IgG binding to AChR & summary graphs, showing patient IgM and IgA binding to AChR. **(A-F)** MG patient autoantibody binding pattern to AchR+Rapsyn transfected cells. Representative images from additional patients in categories 1 and 4, showing AChR expression **(A, C, E)** and patient IgG binding **(B, D, F)**, with nuclear stain in blue, AF647 α-Bungarotoxin in red and Cy3 Anti-Human IgG in yellow. **(A-B)** MG Patient 2 (Category 1), **(C-D)** MG Patient 8 (Category 4), **(E-F)** MG Patient MSDN12 (Category 4). **(G-H)** Image analysis summary graphs, showing background corrected % IgM Positive cells **(G)** and % IgA Positive cells **(H)**. The background was corrected by subtracting the fluorescence of the AChR negative cell population from the fluorescence of the AChR positive cell population. The bars represent the Mean±SEM, n=3 experiments, except for MG Patient 1 where the bars represent the Mean±SD n=2 replicates (n=1 experiment) due to insufficient sample volume to perform additional repeats. Statistical significance was obtained using one-way ANOVA with Dunnet's multiple comparison test, comparing the mean (n=3 experiments) of each plasma sample to the mean of the pooled control plasma sample, except for MG Patient 1 (n=1 experiment, 2 replicates), where statistical significance was obtained using an unpaired t-test, comparing the mean of the two MG patient 1 replicates to the mean of the two pooled control plasma replicates from the same experiment.

S8: Additional patients – Category 1, example 2: weaker binding and lower MAC deposition, but blocking C7 effectively inhibits AChR loss (strong complement role in MG)

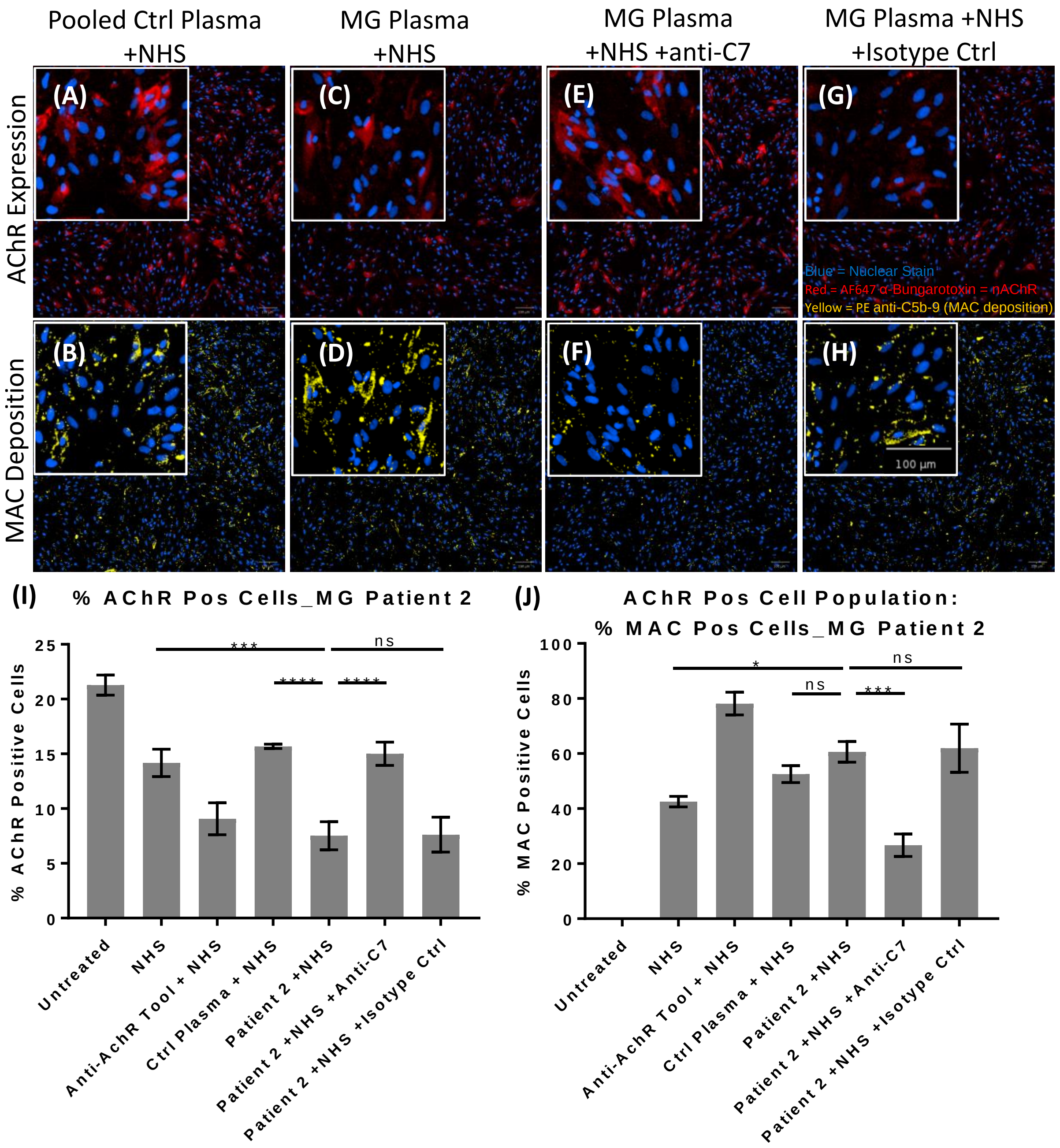

S8: Second example of category 1 MG patient plasma tested in the AChR loss and MAC deposition assay. **(A-H)** Representative images from MG patient 2 showing AChR expression **(A, C, E, G)** and MAC deposition **(B, D, F, H)**, with nuclear stain in blue, AF647 α-Bungarotoxin in red and PE anti-C5b-9 in yellow. “NHS” refers to Ig-depleted NHS in this figure. **(A-B)** Pooled control plasma + NHS, **(C-D)** MG Plasma +NHS, **(E-F)** MG Plasma +NHS +anti-C7, **(G-H)** MG Plasma +NHS +Isotype Ctrl. **(I-J)** Image analysis summary graphs for MG patient 2, showing % AChR positive cells **(I)** and % MAC positive cells within the AChR positive cell population **(J)**. The bars represent the Mean±SEM, n=3 experiments. Statistical significance was obtained using a repeated measures one-way ANOVA without correction, using Tukey's multiple comparisons test.

S9: Additional patients – Category 2 example: undetectable MAC deposition, but blocking C7 still effectively inhibits AChR loss

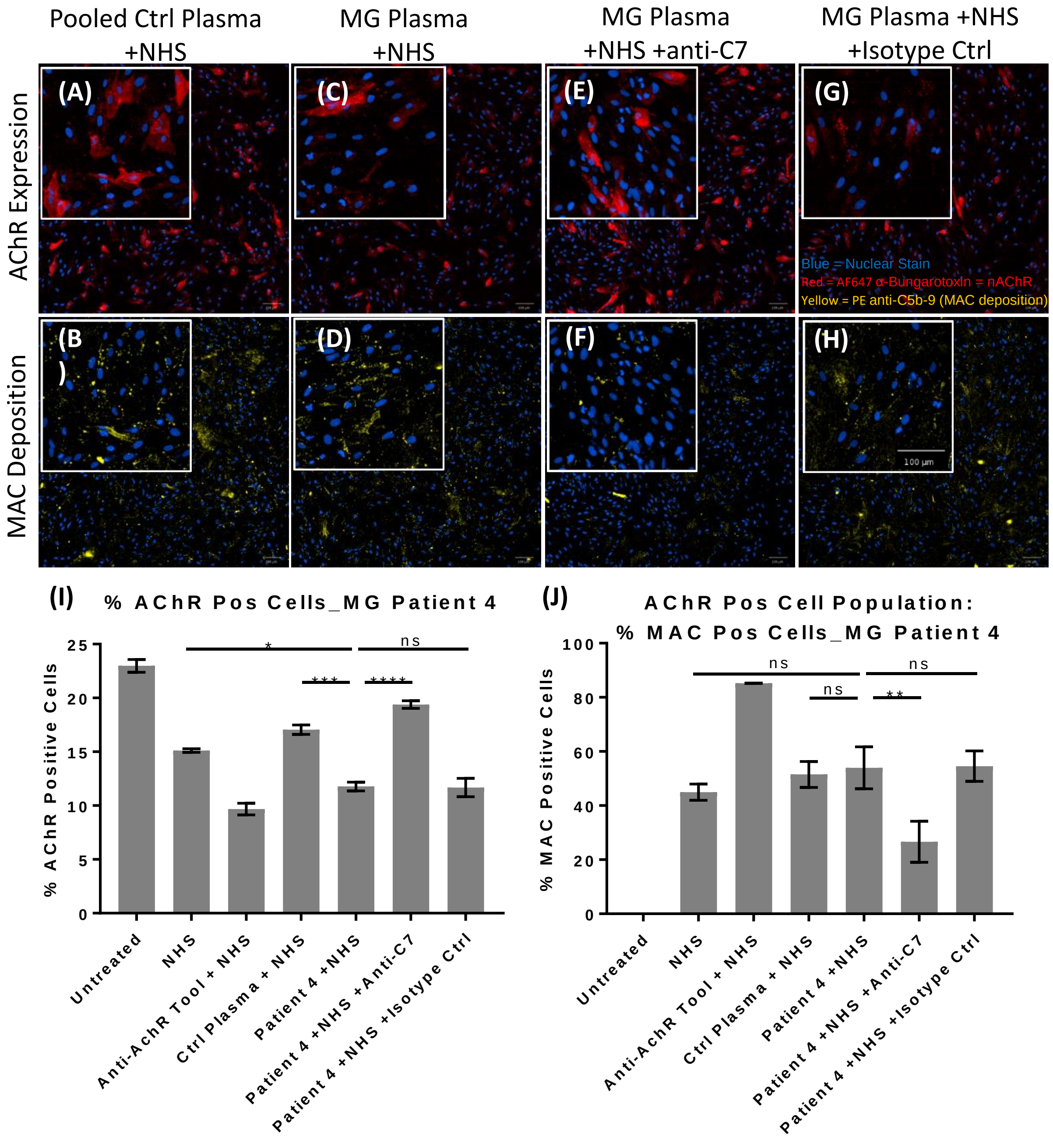

S9: Example of category 2 MG patient plasma tested in the AChR loss and MAC deposition assay. **(A-H)** Representative images from MG patient 4 showing AChR expression **(A, C, E, G)** and MAC deposition **(B, D, F, H)**, with nuclear stain in blue, AF647 α-Bungarotoxin in red and PE anti-C5b-9 in yellow. “NHS” refers to Ig-depleted NHS in this figure. **(A-B)** Pooled control plasma + NHS, **(C-D)** MG Plasma +HNS, **(E-F)** MG Plasma +NHS + anti-C7, **(G-H)** MG Plasma +NHS +Isotype Ctrl. **(I-J)** Image analysis summary graphs for MG patient 4, showing % AChR positive cells **(I)** and % MAC positive cells within the AChR positive cell population **(J)**. The bars represent the Mean±SEM, n=3 experiments. Statistical significance was obtained using a repeated measures one-way ANOVA without correction, using Tukey's multiple comparisons test.

S10: Additional patients – Category 3 example: Binding to AChR but undetectable AChR loss and MAC deposition (no complement role - ligand-blocking antibodies?)

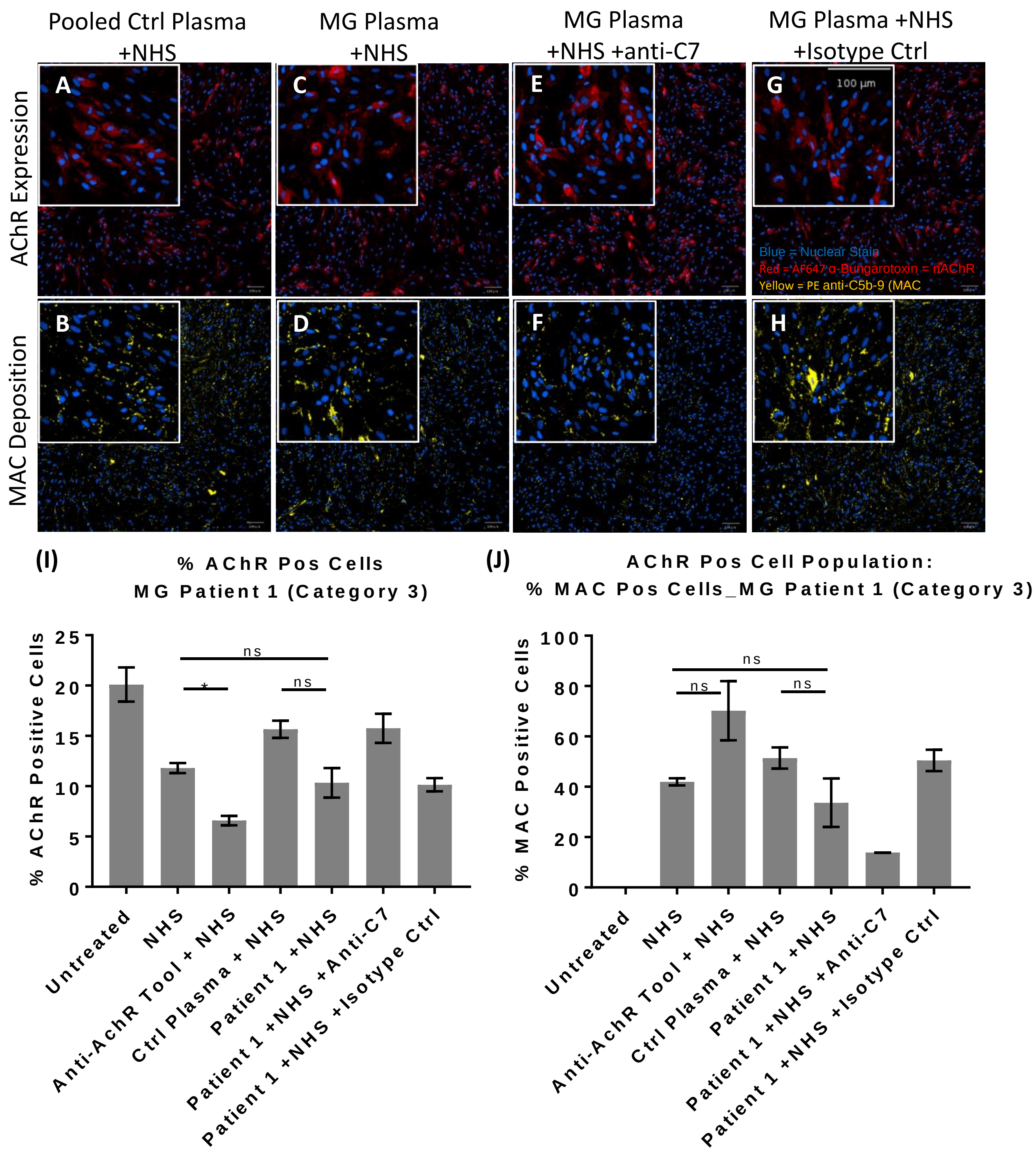

S10: Example of category 3 MG patient plasma tested in the AChR loss and MAC deposition assay. **(A-H)** Representative images from MG patient 1 showing AChR expression **(A, C, E, G)** and MAC deposition **(B, D, F, H)**, with nuclear stain in blue, AF647 α-Bungarotoxin in red and PE anti-C5b-9 in yellow. "NHS" refers to Ig-depleted NHS in this figure. **(A-B)** Pooled control plasma + NHS, **(C-D)** MG Plasma +NHS, **(E-F)** MG Plasma +NHS +anti-C7, **(G-H)** MG Plasma +NHS +Isotype Ctrl. **(I-J)** Image analysis summary graphs for MG patient 1, showing % AChR positive cells **(I)** and % MAC positive cells within the AChR positive cell population **(J)**. The bars represent the Mean±SD, (n=2 replicates from one experiment). Statistical significance was obtained using unpaired t-test.

S11: Additional patients – Category 4, example 1: moderate anti-AChR titre, no IgG cell binding, no AChR loss, no MAC deposition (no detectable complement role in MG – false positive in ELISA?)

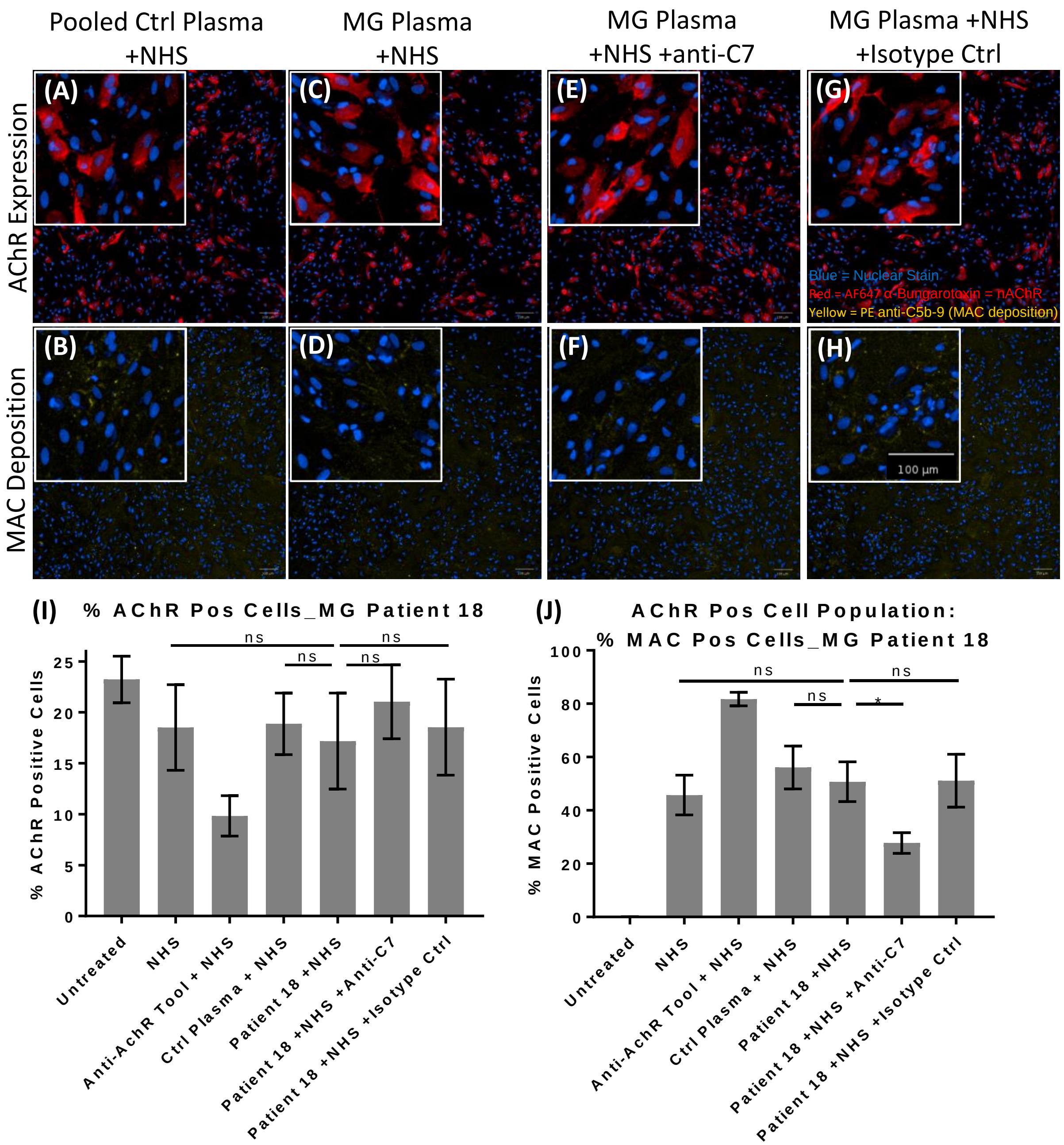

S11: First example of category 4 MG patient plasma tested in the AChR loss and MAC deposition assay. **(A-H)** Representative images from MG patient 18 showing AChR expression **(A, C, E, G)** and MAC deposition **(B, D, F, H)**, with nuclear stain in blue, AF647 α-Bungarotoxin in red and PE anti-C5b-9 in yellow. “NHS” refers to Ig-depleted NHS in this figure. **(A-B)** Pooled control plasma + NHS, **(C-D)** MG Plasma +NHS, **(E-F)** MG Plasma +NHS + anti-C7, **(G-H)** MG Plasma +NHS +Isotype Ctrl. **(I-J)** Image analysis summary graphs for MG patient 18, showing % AChR positive cells **(I)** and % MAC positive cells within the AChR positive cell population **(J)**. The bars represent the Mean±SEM, n=3 experiments. Statistical significance was obtained using a repeated measures one-way ANOVA without correction, using Tukey's multiple comparisons test.

S12: Additional patients – Category 4, example 2: No complement role in MG (MGFA score II), no anti-AChR Abs detectable by either ELISA or cell binding – potential anti-Musk?

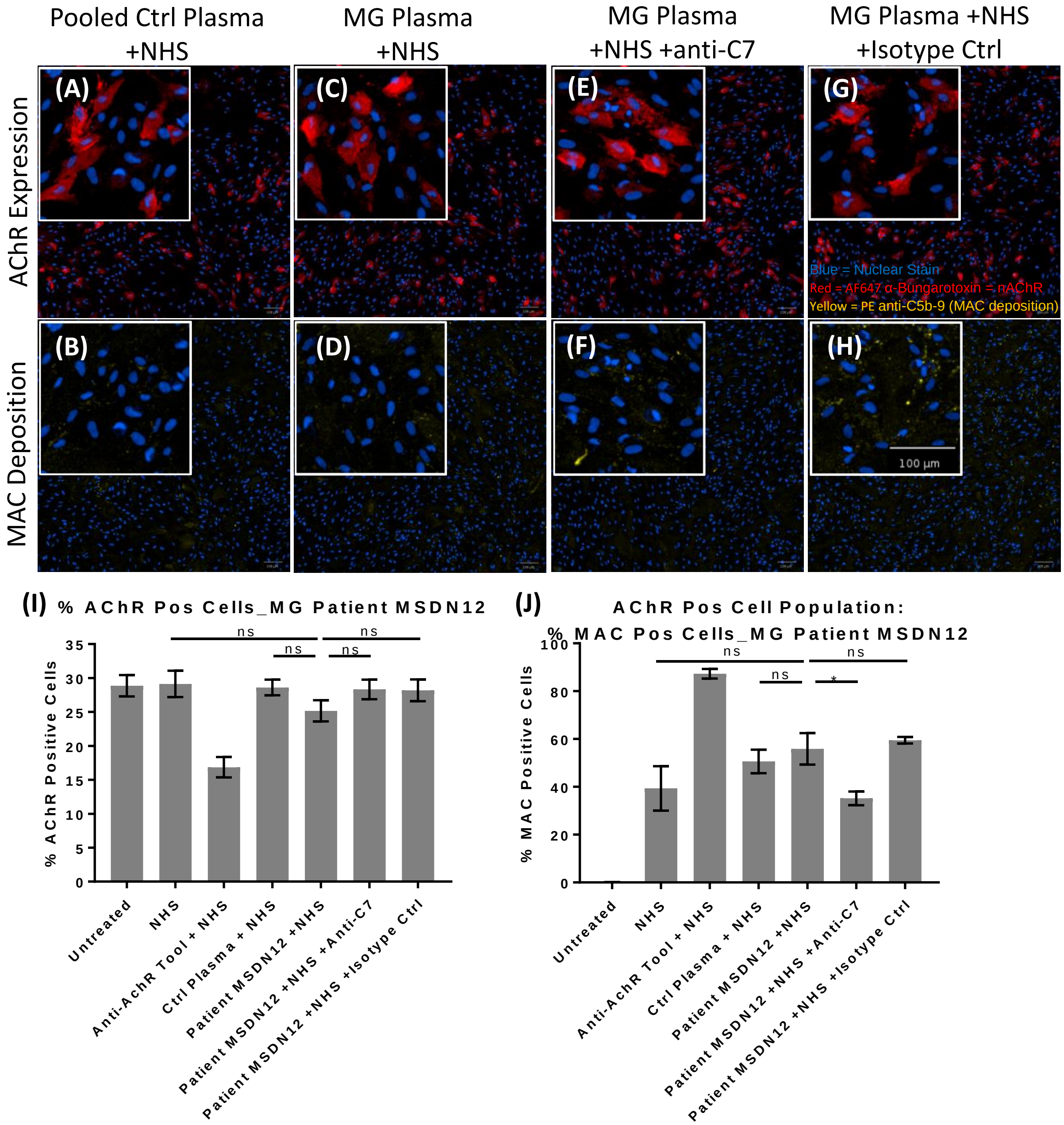

S12: Second example of category 4 MG patient plasma tested in the AChR loss and MAC deposition assay. **(A-H)** Representative images from MG patient MSDN12 showing AChR expression **(A, C, E, G)** and MAC deposition **(B, D, F, H)**, with nuclear stain in blue, AF647 α-Bungarotoxin in red and PE anti-C5b-9 in yellow. "NHS" refers to Ig-depleted NHS in this figure. **(A-B)** Pooled control plasma + NHS, **(C-D)** MG Plasma +NHS, **(E-F)** MG Plasma +NHS + anti-C7, **(G-H)** MG Plasma +NHS +Isotype Ctrl. **(I-J)** Image analysis summary graphs for MG patient MSDN12, showing % AChR positive cells **(I)** and % MAC positive cells within the AChR positive cell population **(J)**. The bars represent the Mean±SEM, n=3 experiments. Statistical significance was obtained using a repeated measures one-way ANOVA without correction, using Tukey's multiple comparisons test.

S13: Additional patients – Category 4, example 3: low anti-AChR titre, low IgG cell binding, no AChR loss, no MAC deposition (below assay detection limits?)

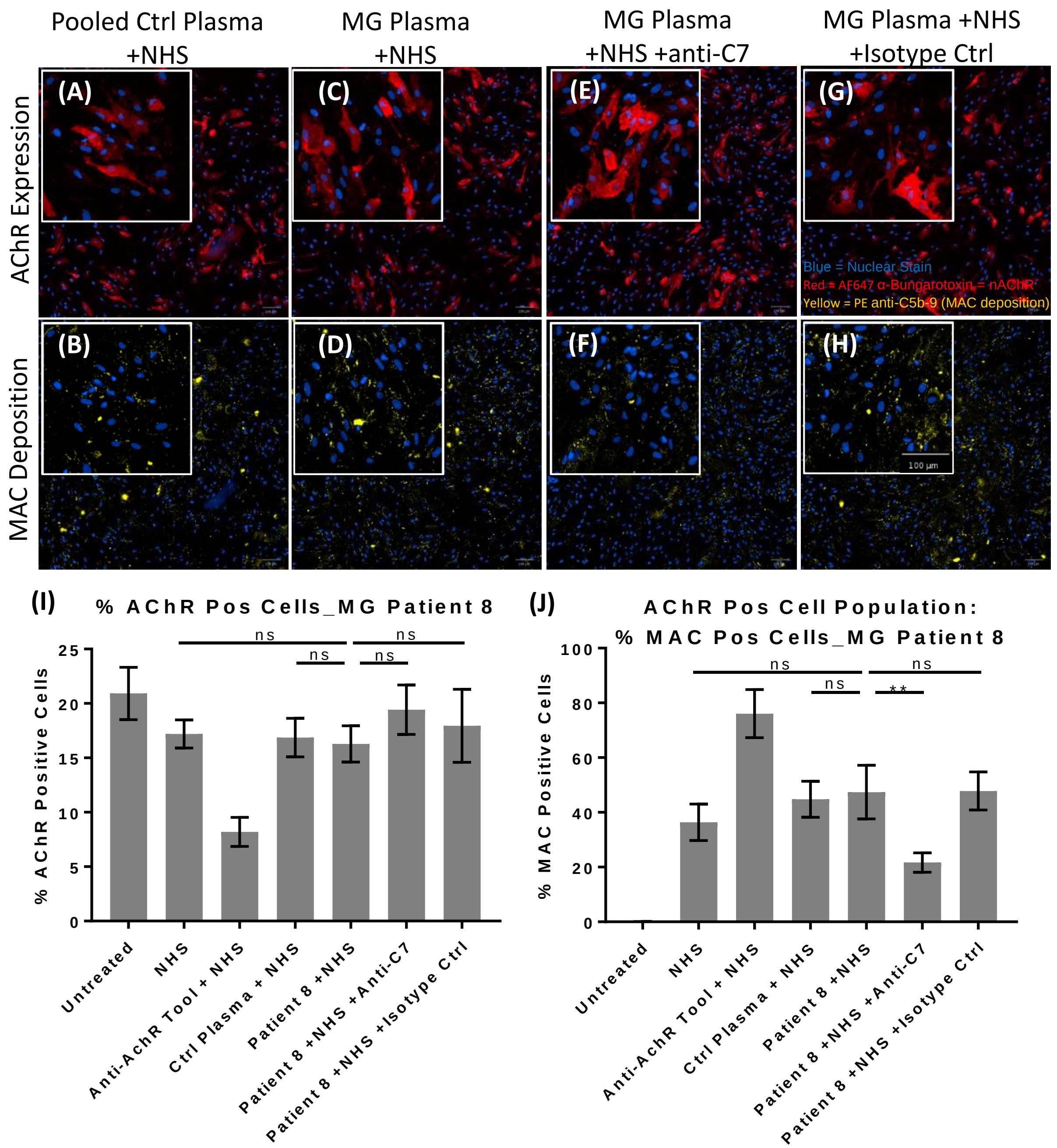

S13: Third example of category 4 MG patient plasma tested in the AChR loss and MAC deposition assay. **(A-H)** Representative images from MG patient 8 showing AChR expression **(A, C, E, G)** and MAC deposition **(B, D, F, H)**, with nuclear stain in blue, AF647 α-Bungarotoxin in red and PE anti-C5b-9 in yellow. “NHS” refers to Ig-depleted NHS in this figure. **(A-B)** Pooled control plasma + NHS, **(C-D)** MG Plasma +NHS, **(E-F)** MG Plasma +NHS + anti-C7, **(G-H)** MG Plasma +NHS +Isotype Ctrl. **(I-J)** Image analysis summary graphs for MG patient 8, showing % AChR positive cells **(I)** and % MAC positive cells within the AChR positive cell population **(J)**. The bars represent the Mean±SEM, n=3 experiments. Statistical significance was obtained using a repeated measures one-way ANOVA without correction, using Tukey's multiple comparisons test.

S14: Correlations between ELISA anti-AChR titres, IgG cell binding to AChR and MAC deposition

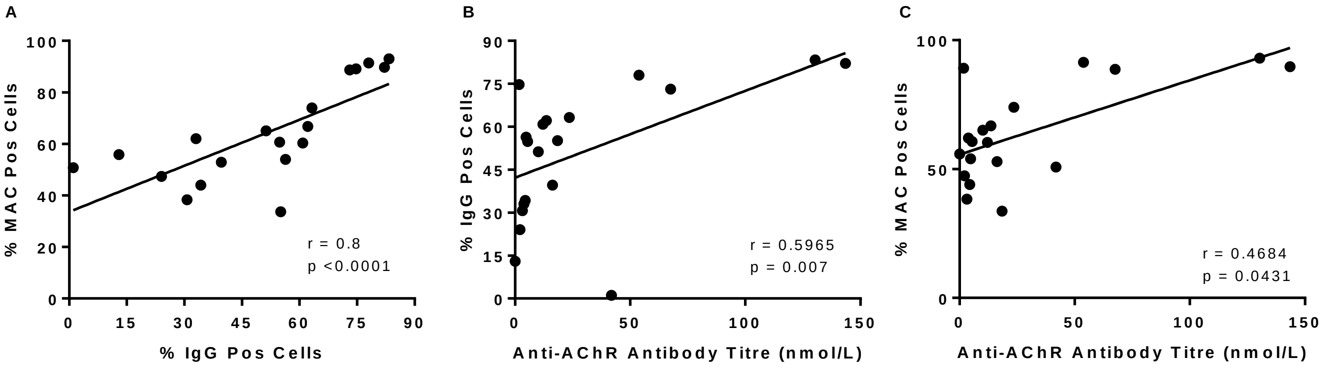

S14: Correlation between the following three data sets: IgG cell binding to AChR vs. MAC deposition on the AChR positive cells (**A**); IgG cell binding to AChR vs. ELISA anti-AChR titres (**B**); ELISA anti-AChR titres vs. MAC deposition on the AChR positive cells (**C**). A two-tailed, non-parametric Spearman correlation with 95 % confidence interval was calculated and the mean values from each pair of data sets being compared were plotted in in GraphPad Prism v7.05

Table S3: Patient details and anti-AChR autoantibody titre

Patient No	Donor ID	Age	Sex	MGFA classification	Anti-Acetylcholine Receptor Antibody (Datasheet)	Anti-Acetylcholine Receptor Antibody (In-House Data)
					nmol/L	nmol/L
1	2032293716	66 year(s) 7 month(s) 29 day(s)	F	Not provided	18,40	Not Tested
2	2032304683	50 year(s) 8 month(s) 23 day(s)	F	Not provided	9,60	5.43
3	2032338964	79 year(s) 6 month(s) 17 day(s)	F	Not provided	270	143.49
4	2032332689	66 year(s) 11 month(s) 29 day(s)	M	Not provided	9.4	4.80
5	2032331318	80 year(s) 4 month(s) 1 day(s)	M	Not provided	3.8	16.28
8	2033025476	87 year(s) 11 month(s) 4 day(s)	M	Not provided	0,56	2.18
9	2033008247	85 year(s) 11 month(s) 15 day(s)	M	Not provided	4,00	3.21
11	2032989947	72 year(s) 9 month(s) 12 day(s)	M	Not provided	6,80	4.37
12	2033030028	53 year(s) 11 month(s) 15 day(s)	F	Not provided	7,40	10.13
13	2032982232	72 year(s) 1 month(s) 29 day(s)	M	Not provided	10,80	3.80
14	2032997019	61 year(s) 10 month(s) 22 day(s)	M	Not provided	13,00	13.65
15	2032972675	57 year(s) 1 month(s) 16 day(s)	M	Not provided	18,50	12.09
16	2032992447	86 year(s) 10 month(s) 23 day(s)	M	Not provided	40,40	23.60
17	2033005700	57 year(s) 3 month(s) 8 day(s)	F	Not provided	62,00	67.52
18	2032974599	40 year(s) 0 month(s) 26 day(s)	F	Not provided	90,50	41.89
MSDN04		58	F	IV	Not provided	130.32
MSDN09		54	M	IV	Not provided	1.78
MSDN12		21	M	II	Not provided	<Detection Range
MSDN19		75	F	IIB	Not provided	53.84
Pooled Ctrl Plasma		Not Known	Not known	N/A	N/A	<Detection Range

Table S4: Correlation Results

	% IgG Pos Cells vs. % MAC Pos Cells	% IgG Pos Cells vs. Anti-AChR Antibody Titre (nmol/L)	% MAC Pos Cells vs. Anti-AChR Antibody Titre (nmol/L)
**r (rs, rho)**	0.8	0.5965	0.4684
**95% confidence interval**	0.5329 to 0.9221	0.1812 to 0.8312	0.003569 to 0.7668
**P value (two-tailed)**	<0.0001	0.007	0.0431
**P value summary**	****	**	*
**Exact or approximate P value?**	Approximate	Approximate	Approximate
**Significant? (alpha = 0.05)**	Yes	Yes	Yes
**Number of XY Pairs**	19	19	19
